# Ecology of an endemic primate species (*Macaca siberu*) on Siberut Island, Indonesia

**DOI:** 10.1186/2193-1801-2-137

**Published:** 2013-03-29

**Authors:** Christin Richter, Ahmad Taufiq, Keith Hodges, Julia Ostner, Oliver Schülke

**Affiliations:** Courant Research Center Evolution of Social Behaviour, Georg-August University Göttingen, Kellnerweg 6, Göttingen, 37077 Germany; Universitas Andalas, Department of Biology, Andalas University, Kampus Limau Manih, Padang, West Sumatra Indonesia; Reproductive Biology Unit, German Primate Center, Kellnerweg 4, Göttingen, 37077 Germany

**Keywords:** Macaque ecology, Species comparison, Southeast Asian rainforest, Endemic species, Conservation

## Abstract

Logging and forest loss continues to be a major problem within Southeast Asia and as a result, many species are becoming threatened or extinct. The present study provides the first detailed and comprehensive ecological data on the Siberut macaque (*Macaca siberu*), a primate species living exclusively on the island of Siberut off the west coast of Sumatra. Our results show that *M. siberu* is ecologically similar to its closest relative *M. nemestrina* occurring on the mainland, both species being semi-terrestrial, mainly frugivorous (75-76%), exhibit a large daily travel distance for their group size and spend more time on traveling than any other macaque species. The habitat of Siberut macaques was floristically very diverse (Simpson’s index D=0.97), although somewhat impoverished in tree species richness, and had a lower tree basal area and a lower rattan density compared to other forests in Malesia (both rattan and palm tree fruit being an important food resource for *Macaca siberu* due to their long fruiting periods). These factors may lead to a lower diversity and abundance of fruit resources, and coupled with a high degree of frugivory of Siberut macaques, may explain the large amount of traveling observed in this species. The large home range requirements and strong dependence on fruit are important factors that need to be considered when developing conservation measures for this IUCN-listed (Category Vulnerable) species.

**Electronic supplementary material:**

The online version of this article (doi:10.1186/2193-1801-2-137) contains supplementary material, which is available to authorized users.

## Background

Tropical rainforests occupy only 7% of our Earth’s land surface but are home to over half of all the species on the planet (Thomas and Baltzer 2002). They are of great importance for the world’s economy and ecology by providing timber, food products and pharmaceuticals, and because they play a major role in the global carbon cycle (also as carbon sinks) and shape local climate patterns (Laurance 1999;Thomas and Baltzer 2002;FAO 2010). While the area of planted forest and conservation efforts are steadily increasing, forest loss and conversion still continue globally at high rates (1990-2000: 16 million ha, 2000-2010: 13 million ha; FAO 2010). Especially in Southeast Asia, which has experienced fast development in the last decades, deforestation rates increased drastically (1880-1980: 0.3%, 1990-1997: 0.91%, 2000-2010: 1%; Flint 1994;Achard et al. 2002;Miettinen et al. 2011), with Indonesia being one of the most critical countries (FAO 2010), and Sumatra among the most critical regions (1985-2007: 48% forest cover loss; Achard et al. 2002;Hansen et al. 2009;Laumonier et al. 2010;Miettinen et al. 2011). While forest loss continues, many of the species existing in these tropical habitats still remain to be discovered or described. The role of these species within the ecosystem is still unknown, and reduction in species diversity may lead to the loss of important services for the ecological community (Díaz et al. 2006).

In this environmentally critical region lies the Mentawai Archipelago, consisting of four small islands 85 to 135 km off the west coast of Sumatra, Indonesia (Whitten 1982c; Fuentes1997/1997;Whittaker 2006). It is part of the biodiversity hotspot Sundaland, which covers Malaysia, Brunei, Singapore and the western half of Indonesia (Mittermeier et al. 1999;Myers et al. 2000;Myers 2001;Mittermeier et al. 2004). Among all 34 biodiversity hotspots worldwide, Sundaland has the highest number of endemic plant species (15,000 - same as Tropical Andes) and the highest number of endemic mammal species (173), of which 81% are already listed as threatened by IUCN (Mittermeier et al. 2004: p. 32-33, 64). This high species richness and endemism in Sundaland cannot be attributed to the amount of habitat alone, as Sundaland only ranks twelfth among all hotspots, with 100,571 km^2^ of moist broadleaf forest (Mittermeier et al. 2004: p. 32). Rather, it is most likely the result of a dynamic geological past of Quaternary glaciations and episodic sea-level changes, during which Sundaland was repeatedly connected to the Asian mainland, enabling species migrations from the mainland to the islands of Sundaland (Gathorne-Hardy et al. 2002;Meijaard 2003;Sodhi et al. 2004;Woodruff 2010;Gower et al. 2012). Additionally, the rise in sea-level and increased isolation of islands which occurred during interglacial periods facilitated the speciation process (Sodhi et al. 2004). Furthermore, during the Pleistocene, some parts of Borneo and the northern and western part of Sumatra, including the Mentawai Islands, acted as rainforest refugia, enabling the survival of these rainforest biota (Gathorne-Hardy et al. 2002;Meijaard 2003).

The Mentawai Islands, of which Siberut is the largest and northernmost island, have been separated from the mainland for over 0.5 m years (Mitchell and Tilson 1986;Voris 2000;Bird et al. 2005). Even at times when sea level was so low that the rest of Sundaland was connected, the 1,700 m deep Mentawai Basin maintained the separation of the islands from the mainland (Brune et al. 2010). In fact, the Mentawai Islands were never connected to Sundaland directly, but were linked to Sumatra via a land bridge from Siberut through the Batu Islands (Whittaker 2006). As a result of this long period of geographic isolation, the Mentawai Archipelago has evolved a distinct flora and fauna with a high level of endemism, and allowed the survival of a number of “primitive” forms of considerable evolutionary interest (WWF 1980).

The flora of Siberut is estimated to comprise about 15% endemic plant forms, but the figure is out of date and new research is needed (WWF 1980). Of those species also known from other areas in Southeast Asia, some developed distinct traits on Siberut (WWF 1980: p. 13). The Mentawaian fauna includes 43 mammal species, of which 42% are endemic to Mentawai, and without bats, the endemism level increases to 71% (Thorington Jr. et al. 2012;Wilting et al. 2012). That the fauna of Siberut still remains understudied was recently shown by Kemp (2000), who recorded 28 new bird species for the island.

The ecosystems on small isolated islands such as Siberut are usually shaped by a range of different factors: Firstly, small islands often have an impoverished flora and fauna compared to the mainland, since species richness has been shown to decrease with land area (MacArthur and Wilson 1967;Simberloff 1974;Heaney 1984;Burkey 2002;Kreft et al. 2008;Nijman and Meijaard 2008). Usually the poorly dispersing species or large animals with large home range requirements are absent on small islands (Simberloff 1974;Heaney 1984). Secondly, a lack of certain species or whole taxa is usually associated with increased density of a few other species (density compensation) and a broader niche of island species compared to their relatives on the mainland (niche expansion), where competition for the same resources is higher (MacArthur et al. 1972;Buckley and Jetz 2007;Yoder et al. 2010). Niche expansion or niche shifts between islands and the mainland can concern habitat, vertical foraging strata, altitudes, foraging techniques and diet (MacArthur et al. 1972;Yoder et al. 2010). On Siberut, such niche expansion has been demonstrated for the spangled drongo (*Dicrurus hottentotus*) and three squirrel species (: Whitten*Callosciurus melanogaster, Sundasciurus (lowii) fraterculus, Lariscus obscurus*^1981^;Whitten 1982e). On islands, fewer species compete for the same niche, so that evolutionary pressure becomes lower and populations or species evolve less rapidly. Thus, more “primitive” (archaic) forms can be maintained than on the mainland (WWF 1980;Patou et al. 2010). Despite these general trends geographical isolation can also lead to the evolution of new forms (Yoder et al. 2010).

Primate species richness on Mentawai is unusually high. On a land surface area of only 6,549 km^2^, Mentawai harbors five endemic primate species (Fuentes1997/1997), the Kloss gibbon (*Hylobates klossii*), the Mentawai langur (*Presbytis potenziani*), the pig-tailed langur or pig-tailed snub-nosed monkey (*Simias concolor*), the Pagai island macaque (*Macaca pagensis*) and the Siberut macaque (*Macaca siberu*). Whereas the first three species occur on all four islands, *M. pagensis* only occurs on the three southern islands, and *M. siberu* exclusively on Siberut. All Mentawaian primates are included in the IUCN Red List of Threatened Species (IUCN 2012). They are threatened by habitat loss due to legal and illegal logging, conversion of the forest into oil palm plantations, forest clearing, extraction of forest products (such as rattan), hunting and pet trade (Whittaker 2006). In Siberut, forest cover has decreased to 60% (by 2005; Whittaker 2006), but part of it is protected by the national park, which includes 465 km^2^ of protected “no-use” sanctuary zone where no hunting and logging is allowed (WWF 1980; Fuentes1997/1997;Whittaker 2006).

Of the four primate species occurring on Siberut, the Kloss gibbons, the Mentawai langurs and the pig-tailed langurs have been reasonably well studied (e.g. Tenaza 1975;Tilson 1977;Watanabe 1981;Tilson and Tenaza 1982;Whitten 1982a;Whitten 1982b;Whitten 1982c;Tenaza and Tilson 1985;Fuentes 1997;Hadi et al. 2009a;Erb et al. 2012b;Hadi et al. 2012). In contrast, studies on the Siberut macaque are mainly limited to investigations on population size, acoustic traits, phylogenetic relationships and some preliminary observations on ecology (Whitten and Whitten 1982;Abegg and Thierry 2002b;Roos et al. 2003;Ziegler et al. 2007;Schneider et al. 2008;Waltert et al. 2008;Quinten et al. 2009). Initially, Siberut macaques were thought to be a subspecies of southern pigtail macaques ( Fooden*Macaca nemestrina pagensis:*^1975^) or of the Mentawai/ Pagai macaque of the southern Mentawai Islands (*Macaca pagensis siberu:* Fuentes and Olson^1995^;Groves 1997). Recently, however, genetic and morphological studies have allowed their classification as a distinct species called *M. siberu* (Kitchener and Groves 2002;Roos et al. 2003), being more closely related to *M. nemestrina* on Sumatra and Malaysia than to *M. pagensis* on the neighboring Mentawai Islands (Ziegler et al. 2007). So far, no detailed and comprehensive systematic behavioral or ecological studies have been conducted on Siberut macaques.

The present study aimed to gather basic ecological knowledge about the endemic Siberut macaques, including home range requirements, habitat and forest structure use and feeding habits. The data will add substantially to what is known about the ecological range of the genus *Macaca*, which is among the most successful non-human primate genera with more than 20 species distributed from North Africa throughout Asia up to Japan (Abegg and Thierry 2002a). Siberut macaques, as part of the *silenus-sylvanus* lineage, are thought to represent a relict species from the earliest wave of macaque dispersal and thus may also help to understand the ancestral traits of this genus (Fooden 1980;Abegg and Thierry 2002a;Roos et al. 2003;Ziegler et al. 2007). We compared the new data on Siberut macaques with available data from other macaque species, in order to understand the range of ecological variations, and with data on Siberut’s other primate species with the aim of investigating possible niche differentiations. In addition, we present data on the habitat of Siberut macaques to investigate whether the forest is impoverished as island biogeography theory would predict, which could have important impacts on the behavior of Siberut macaques. The data should also be useful in the development of much needed conservation guidelines for the species.

## Results

### Home range size and daily travel distance

**Figure 1:**
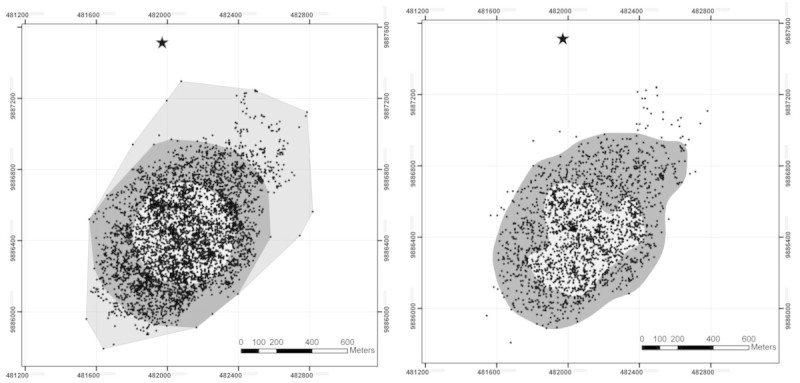
**Home range areas of the study group.** Left: 100%, 95% and 50% minimum convex polygons (MCP’s). Right: 95% and 50% contour of the fixed kernel home range using reference bandwidth. The star is indicating the research station and the cloud of small dots represents the locations of group scan observations. Grid shows UTM coordinates (UTM Zone 47 South). One grid square equals 400x400m.

The total area used by the group during one year was 134.9 ha, based on 100% MCP. When excluding outliers, the area was 80.6 ha (95% MCP), with a core home range of 26.1 ha (50% MCP). Using the fixed kernel method with reference bandwidth, the 95% contour included 84.1 ha, and the 50% contour 26.2 ha (Figure 1). Excluding days with short contact times (<6 h per day) did not change the shape or the size (average change of 0.1 ha) of the home ranges. Home range size plateaued at ~900 fixes, i.e. 5 full months of observation.

**Figure 2:**
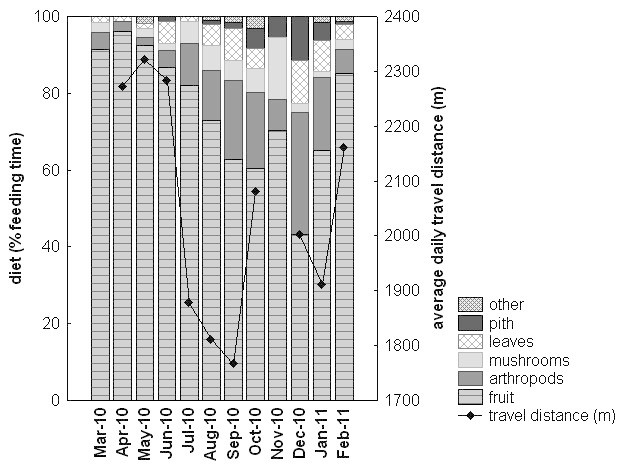
**Diet and average daily travel distance per month.** Diet is calculated as percent feeding time on fruit, arthropods, mushrooms, leaves (mainly young leaves and young leave petioles), pith (the soft core of the palm stem) and other food items, which includes flowers, sap and shoots. Data on travel distance for March 2010 and November 2010 were omitted because they were too scarce.

**Figure 3:**
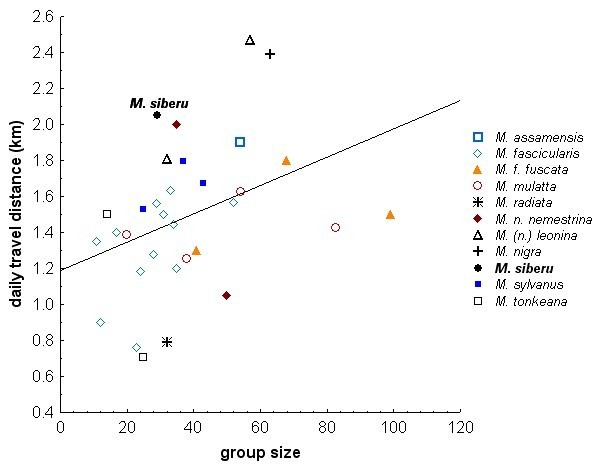
**Mean daily travel distances (in km) of free-ranging macaque groups and species in undisturbed habitats unless otherwise stated.** Sources and study sites: *M. assamensis*: Schülke et al (2011), Phu Kieo Wildlife Sanctuary, Thailand; *M. fascicularis*: 3 groups from C. Girard-Buttoz (pers. comm.), Ketambe, Sumatra, tropical lowland evergreen forest, Mar. ’10 – Apr. ’11, data from 2 adult males per group; 7 groups (A, G, K77, H77, K, H, T, A) from van Schaik et al (1983), Ketambe, Sumatra, 1 group (A) from Aldrich-Blake (1980), Kuala Lompat, W-Malaysia; 1 group from MacKinnon & MacKinnon (1980), Kuala Lompat, W-Malaysia; *M. f. fuscata*: data from the studies of Ikeda (1982) at Kawaradake, Japan, Wada (1979), Shiga heights, Japan, and Izumiyama (1999), Kamikochi, Japan, as in Tsuji (2010); *M. mulatta*: 3 groups (B, C, E) from Neville (1968), Uttar Pradesh, N-India, 1 group (Asarori II) from Lindburg (1971) and Lindburg (1977: Sugiyama (), Uttar Pradesh, India; *M. radiata*^1971^: Caldecott (), Dharwar, S-India; *M. n. nemestrina*^1986^a), Lima Belas, W-Malaysia, forest surrounded by oil palm plantations; MacKinnon & MacKinnon (1980), Kuala Lompat, W-Malaysia; *M. (n.) leonina*: HQ troop from Albert (2012), Khao Yai National Park, Thailand, close to human settlement, only day range data used from when the group was not using human food (high fruit abundance time); forest group (Ch troop) from J. M. José Domínguez (pers. comm.), Khao Yai National Park, Thailand, May – Jun. ’11 and May – Aug. ‘12; *M. nigra*: O’Brian & Kinnaird (1997), Tangkoko, Sulawesi; *M. siberu*: this study; *M. sylvanus*: 2 groups from Ménard & Vallet (1997) at Djurdjura and Akfadou, Algeria; 1 group (group 6) from Deag (1974), Ain Kahla, Marocco; *M. tonkeana*: 2 groups from Pombo et al (2004), Lore Lindu National Park, Sulawesi, 1 group in disturbed forest.

The average travel speed of the group (from group scan data) was 169.6 m/h (SD ± 35.6 m/h, range: 87.8 m – 280.0 m, N=78 days), the average travel speed of single adult individuals (from focal observations) was 206.1 m/h (SD ±143.9 m/h, N=120 focal follows). The monthly average daily travel distance of the group equaled 2,048.4 m (SD ±205.5 m, N= 10 months with 6-9 “full day follows” per months), with travel distances of single days ranging between 1,054 m and 3,360 m. Monthly average daily travel distances tended to increase with the percentage of fruit in the diet (r_s_= 0.64, N=10, p=0.055; see Figure 2). The monthly average daily travel distance was 303 m more than needed to cross the 100% MCP home range at its widest point. The largest observed distance moved between two consecutive group scan observations was 657 m within 30 min, and the largest distance moved of a single individual during a focal observation was 542 m in 67 min (by an adult male). Compared to other macaque species, Siberut macaques traveled more per day than other macaque species of similar group size, but they were similar to *Macaca nemestrina*, to whom they are genetically most closely related (Figure 3). Overall, group size was significantly correlated with the distance traveled per day in this comparative data set (Spearman rank: r_s_ = 0.46, N=32, p<0.01).

### Habitat and forest structure use

**Figure 4:**
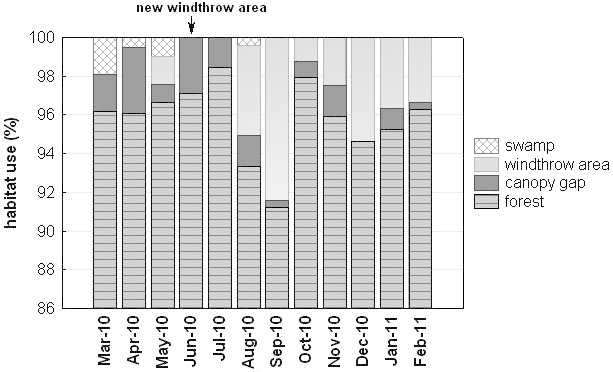
**Percentage of habitat used, defined as forest, canopy gap, windthrow area and swamp.** Windthrow areas were created by heavy storms, one in May 2009 (~4.5 ha, at the SW edge of the home range), and another one beginning of June 2010 (~0.9 ha, within the home range).

Siberut macaques spent most of their time in the dense, continuous forest (average over all months: 95.7%), and used windthrow areas only 2.6%, canopy gaps 1.4%, and swamp areas 0.3% of their time. However, as can be seen in Figure 4, variation across months was high. The two windthrow areas were created by storms in May 2009 (before observations commenced) and June 2010. The use of windthrow areas and swamp were both correlated to the amount of fruit in the diet but in opposite ways. While windthrow areas were used more when less fruit was consumed (r_s_= -0.67, N=12, p=0.02), time spent in swamp areas was positively related to the proportion of fruit in the diet (r_s_ = 0.69, N=12, p=0.02). The former effect may result from generally reduced fruit availability after the storm that created the second windthrow area while the latter effect suggests that swamps are only visited after sufficient amounts of fruit have been ingested.

**Figure 5:**
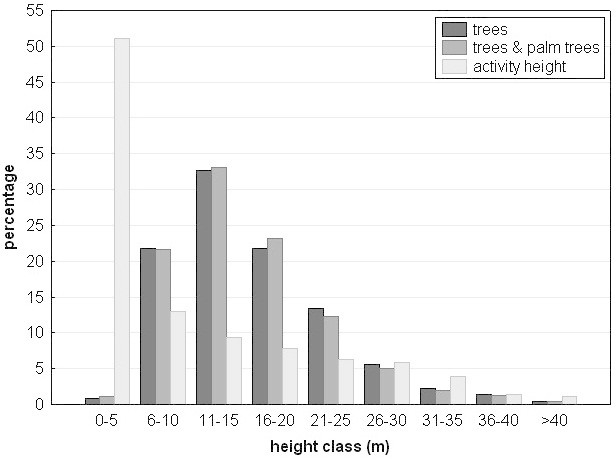
**Distribution of height classes (in m) of trees, trees and palm trees combined and the percentage of the daily daytime activity of the group of Siberut macaques per height class shown.** Tree data are based on all 12 botanical plots.

**Figure 6:**
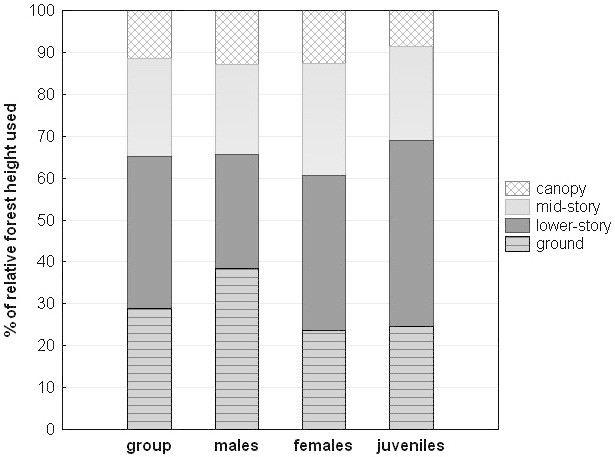
**Percent frequency of the relative forest height used (ground, lower-story, mid-story, canopy) for the whole group, and for adult males, adult females and juveniles separately.**

In terms of the absolute forest height, Siberut macaques mainly used the lower strata of the forest (64.9% of their day-time activities in 0-10 m height, with 53.0% being between 0-5 m), and only to a much lesser extent the upper forest strata (16.5% in 11-20 m, 11.5% in 21-30 m, 5.8% in 31-40 m, and 1.3% in >41 m; see also Table 1 and Figure 5). Regarding the relative height of the forest, we find that the group spent 28.9% of their time on the ground, 36.2% in the lower-story, 23.5% in the mid-story, and only 11.4% in the canopy (Figure 6). Adult males spent more time on the ground than adult females and juveniles. The predominant activities on the ground were traveling (80.2%) and to some extent foraging (10.1%), with feeding, resting and social behavior only accounting for small proportions. Compared to other forest-living macaques in the tropics and subtropics, Siberut macaques fall on the side of more terrestrial macaque species (Table 2) and are more terrestrial than *M. nemestrina* in W-Malaysia (longterm study data), but similarly terrestrial than *M. nemestrina* in Sumatra (only survey data).

**Table 1:** **Comparison of Siberut’s sympatric primate species**

	***S. concolor***	***P. potenziani***	***H. klossii***	***M. siberu***
Group size	2-12	2-8	2-6	29
Home range size	4-20	25-40	5-35	135
(100% MCP, ha)				
Mean day range (m)	572	774	1,508^b^	2,048^c^
(range)	(189-1,200)^a^	(427-1,400)^a^	(885-2,150)	(1,054-3,360)^c^
Diet (%)^d^				
Fruits	22.8	55.4 (32)	72.0	75.7
Flowers	17.8	5.1	0	0.2
Leaves	57.2	34.6 (55)	2.0	4.4
Arthropods	0.6^a^	0.0	25.0	11.9
Other	1.7	4.8 (13)	0	7.8
Activity budget (%)				
Travel/ move	6.2	6.9	11	57.3
Feed	30.8	35.3	34	10.1
Forage	2.4	4.9		12.1
Rest	55.4	50.8	54	14.6
Social	2.3	0.7	x	5.9
Other	2.8	1.4	2	
Forest strata use (%)^e^				
0-10 m	13	14		64
11-20 m	58	52		17
21-30 m	28	33		12
>30 m	1	1		7

Diet expressed as percent feeding time.

^a^ from S. Hadi (pers. comm.), ^b^ median range not mean range from Whitten (1982c), ^c^ mean day range is monthly average for 10 months, range means minimum and maximum of all single “full day follows” during this period, ^d^ diet for *P. potenziani* from Hadi et al (2012), with values from Fuentes (1997) in brackets, ^e^ forest strata use for the three species was recorded at the same study site of the Siberut Conservation Programme, in the Peleonan Forest, North Siberut.

: Erb (Data sources: *Simias concolor*^2012^), Erb et al (2012b), Hadi (2012), Hadi et al (2012) and Tenaza & Fuentes (1995: Fuentes (); *Presbytis potenziani*^1997^), Hadi (2012) and Hadi et al (2012: Tenaza (); *Hylobates klossii*^1975^), Tilson (1981), Whitten (1980), Whitten (1982a), Whitten (1982c); *Macaca siberu*: this study.

**Table 2:** **Percent terrestriality of different free-ranging macaque groups and species, sorted by increasing terrestriality and species**

Species	Study site	Study period	% terrestrial	Source
**Tropical/subtropical climate**				
*M. silenus*	Western Ghats, India	1 year	0.4	1
*M. silenus*	Western Ghats, India	Sep '90 - Aug '91	4.9	2
*M. (n.) leonina*	Bherjan, E-India	1992-'94, 2004	1.5	3
*M. (n.) leonina*	Khao Yai NP, Thailand	Jul - Aug '12	48.5	4
*M. (n.) leonina*	Khao Yai NP, Thailand	Apr '09 - Nov '10	60.0	5
*M. fascicularis*	Kuala Lompat, W-Malaysia	Jul '74 - Jan '76	1.7	6
*M. fascicularis*	Kuala Lompat, W-Malaysia	Jan - Jul '73	2.0*	7
*M. fascicularis*	Sumatra, Indonesia		4.0*	7
*M. fascicularis*	Kutai NR, Kalimantan	Oct '74 - Jun '76	5.0	8
*M. assamensis*	Phu Khieo WS, Thailand	Jul '06 - Jun '07	10.0	9
*M. n. nemestrina*	Lima Belas, W-Malaysia	Jan '80 - May '81	9.0^a,b^	10
*M. n. nemestrina*	Lima Belas, W-Malaysia		15.0^c^	11
*M. n. nemestrina*	Sumatra, Indonesia		25.0^c,^*	7
*M. n. nemestrina*	Sumatra, Indonesia	Nov '71 - Jan '73	>30%^c^	12
*M. cyclopis*	Yushan NP, Taiwan	Mar '87 - Oct '88	15.4*	13
*M. nigrescens*	Dumoga-Bone NP, Sulawesi	Apr '89 - Jun '90	17.3*	14
*M. s. sinica*	Polonnaruwa, Sri Lanka	Sep '68 - early '72	24.1	15
***M. siberu***	Siberut island, Sumatra	Mar '10 - Mar '11	**25.4**	16
*M. radiata*	Dharwar, S-India	Mar - Sep '62	30.0	17
*M. nigra*	Tangkoko, Sulawesi	Jan '93 - Jun '94	>60	18
*M. nigra*	Tangkoko, Sulawesi	Jul '06 - Jun '07	72.0*	19
*M. nigra*	Tangkoko, Sulawesi	Jul '06 - Jun '07	76.7*	19
**Temperate climate**				
*M. f. fuscata*	Takagoyama Area, Japan	1970 - '71	51.2^d^	20
*M. f. fuscata*	Tsubaki, Japan	Jun '95 - Jan '97	54.1	21
*M. sylvanus*	Akfadou, Algeria	Apr '83 - Feb '85	58.4^e^	22
*M. sylvanus*	Djurdjura, Algeria	Apr '83 - Feb '85	68.5^e^	22
*M. mulatta*	N-India (Kaluwala)	1981 - '86	61.4	23
*M. mulatta*	Murree hills, NW-Pakistan	1978 - '79	66.0	24
*M. mulatta*	N-India (Nagal Check Post)	1981 - '86	71.7	23

Terrestriality is defined as time spent on the ground, unless otherwise stated.

Abbreviations of study sites: NP = National Park, NR = Nature Reserve, WS = Wildlife Sanctuary.

^a^ 0-2 m height, ^b^ mean of spot observations and activity assessment, ^c^ survey data, monkeys not really habituated, ^d^ only data for autumn and winter, ^e^ less than 1 m height, * values estimated from figure.

Sources and habitat type: 1) Kurup & Kumar (1993), undisturbed wet evergreen forest; 2) Menon & Poirier (1996), disturbed forest; 3) Choudhury (2008), tropical wet evergreen forest and decidious plantations; 4) J. M. José Domínguez (pers. comm.), seasonal wet evergreen forest, forest group Ch; 5) Albert et al (2011), seasonal wet evergreen forest, close to the national park’s visitor center, group HQ; 6) Aldrich-Blake (1980), tropical lowland evergreen forest; 7) MacKinnon & MacKinnon (1980), site at Kuala Lompat: tropical lowland evergreen forest, site in Sumatra: habitat type not given by the authors; 8) Wheatley (1980), mixed lowland forest; 9) Schülke et al (2011), dry evergreen forest; 10) Caldecott (1986a), tropical broadleaf evergreen forest surrounded by oil palm plantations, value is the mean of spot observations and activity assessments; 11) Bernstein (1967), tropical broadleaf evergreen forest, same study site as Caldecott (1986a); 12) Crockett & Wilson (1980), swamp, lowland, hill and submontane forest; 13) Lu et al (1991), mainly primary broadleaf forest; 14) Kohlhaas (1993), primary lowland rainforest, with some patches of secondary growth and grasses, 15) Dittus (1977), semi-evergreen forest, dry zone plain; 16) this study; 17) Sugiyama (1971), dry decidious forest; 18) O’Brian & Kinnaird (1997), some parts disturbed forest; 19) Giyarto (2010), Rambo I group (1^st^ value) mainly primary forest, Rambo II group mainly secondary forest; 20) Yotsumoto (1976), secondary broadleaf decidious forest, some parts with broadleaf evergreen trees; 21) Chatani (2003), evergreen forest less than 5 m high; 22) Ménard & Vallet (1997), temperate decidious oak forest at Akfadou, temperate evergreen cedar-oak forest at Djurdjura; 23) Chopra et al (1992), forest; 24) Goldstein & Richard (1989) and Goldstein (1984), temperate mixed coniferous decidious forest with disturbed areas.

### Activity budget

**Figure 7:**
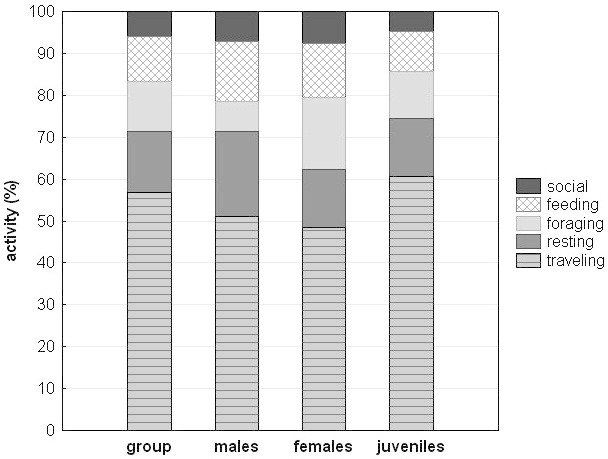
**Activity budget based on group scan data, for the whole group, and for adult males, adult females and juveniles separately.**

**Figure 8:**
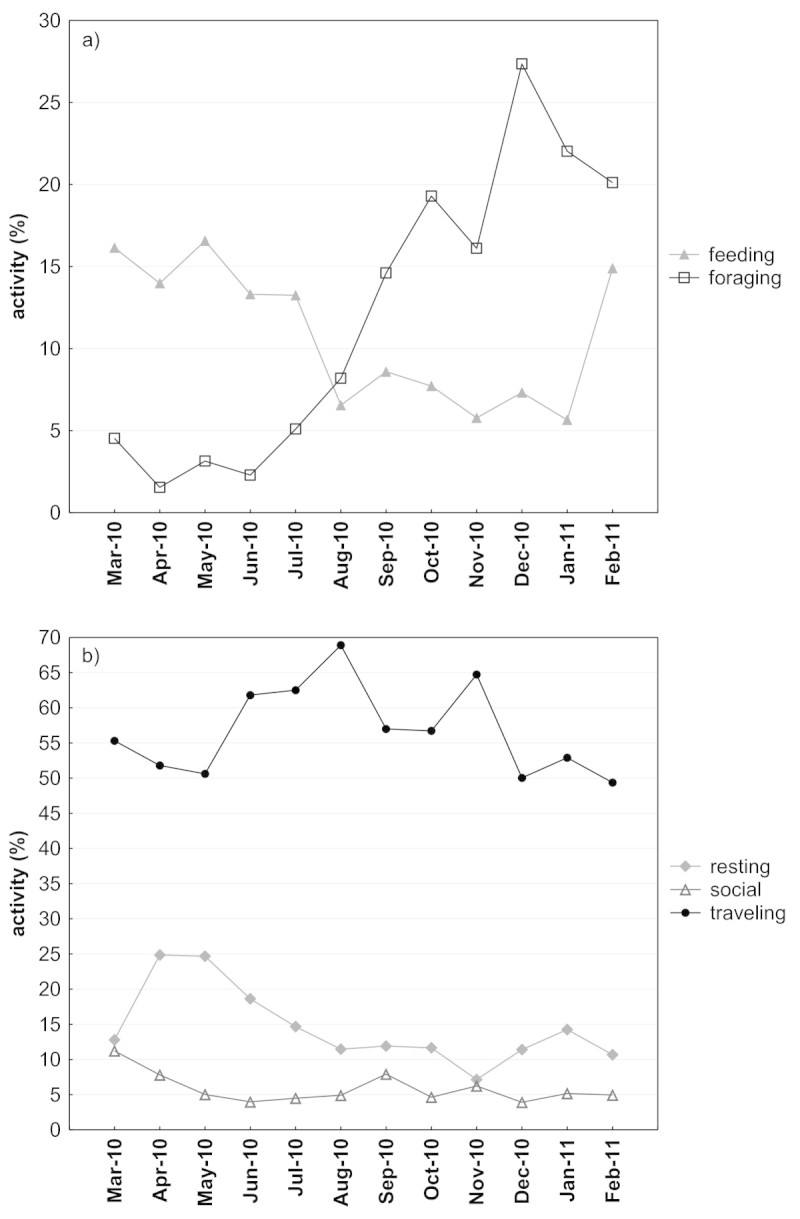
**Variation in the activity budget (in percent) regarding a) time spent feeding and foraging and b) time spent resting, traveling and being social.**

The activity budget revealed that the group spent most of its time traveling, with a monthly average (±SD) of 57.3% (±6.6), 14.6% (±5.6) resting, 12.1% (±9.0) foraging, 10.1% (±4.0) feeding, and only 5.9% (±2.2) on social activities (Figure 7). There was a significant sex-difference in resting (Mann-Whitney-U: W=18, p=0.02), with adult males resting 6.4% more than their female counterparts. There was also a significant sex-difference in time spent foraging (Mann-Whitney-U: W=0, p=0.02), with females spending nearly two and a half times more on searching for food then males (Figure 7, Fisher’s Omnibus Test to control for multiple testing: X^2^=19.6, df=10, p=0.03). For feeding, traveling and social activities, males and females did not differ significantly. The most striking differences between juveniles and adults were the much higher amount of time spent traveling in juveniles (juveniles: 60.7%, females: 48.4%, males: 51.1%), a lower amount of time spent feeding (juveniles: 9.5%, females: 12.9%, males: 14.5%), and only very little time spent on social behavior (juveniles: 4.8%, females: 7.6%, males: 7.1%), which is surprising as playing is part of this category. However, these differences cannot be tested for significance as the majority of the juveniles were not identified. Most of the activities showed large variations throughout the months, and only social behavior was relatively stable (~5%, Figure 8). High variations in feeding time are probably driven by fruit availability in the forest, and as feeding time decreases, the time spent foraging (searching for food) increases, along with an increase of time spent traveling, both at the cost of time spent resting (Figure 8).

Compared to other macaque species, Siberut macaques spent more time traveling (Table 3), which is surprising, given their small group size, but is in accordance with the long daily travel distance observed (Figure 3). Among the 15 species examined in Table 3, only *M. nemestrina* travels more than Siberut macaques. Due to the large amount of time they have to devote to traveling, they only have very little time left for social activities (Table 3).

**Table 3:** **Activity budgets (in percent) of free-ranging and unprovisioned macaque groups of different species, sorted by decreasing travel time and species**

Species	Study site	Study period	Group size (name)	Travel/ Move	Forage	Feed	Rest	Social	Other	Source, Obs.M.
**Tropical/subtropical climate**										
*M. n. nemestrina*	Lima Belas, W-Malaysia	Jan '80 - May '81	50	61.0	16.0		19.0	4.0		1, O
***M. siberu***	Siberut island, Sumatra	Mar '10 - Mar '11	29	57.3	12.1	10.1	14.6	5.9		2, S
*M. silenus*	Western Ghats, S-India	Aug '94 - Mar '96	23	43.6	31.0		20.7	5.8		3, S
*M. silenus* ^*a*^	Anamalai WS, W-Ghats, India	Sep '90 - Aug '91	41-43	34	23.7	17.9	16	8.4		4, S
*M. silenus*	Anamalai WS, W-Ghats, India	1 year	12-31	15	26.7	27.8	27	2.4	1.1	5, S
*M. tonkeana* ^(a),*^	Lore Lindu NP, Sulawesi	Jun '02 - Apr '04	26-28 (Ch)	36.0	7.5	13.0	32.5	11.0		6, S
*M. tonkeana* ^a,*^	Lore Lindu NP, Sulawesi	Jun '02 - Apr '04	6-9 (Anca)	29.0	10.0	14.0	36.0	11.0		6, S
*M. tonkeana*	Lore Lindu NP, Sulawesi		14	31.0		19.3	35.0	14.7		7, S
*M. tonkeana* ^a,*^	Lore Lindu NP, Sulawesi		25	17.7		11.4	51.0	19.9		7, S
*M. (n.) leonina* ^*b*^	Khao Yai NP, Thailand	Apr '09 - Nov '10	32-39 (HQ)	34.0	5	13.0	30.0	16.0	2.0	8, S
M. (n.) leonina *	Bherjan, E-India	1992-'94, '04	20-23 (2 groups)	19.5	23.5		45.0	8.0	4.0	9, S
*M. assamensis*	Chiang Rai, Thailand		x	27.2	16.8		31.2	24.8		10, U
*M. assamensis* ^*c*^	Makalu-Barun NP, Nepal	Mar - Apr '97 + '98	27 (Wa.), 13 (Sa.)	27.0	45.5		16.5	11.0		11, S
*M. assamensis* ^*c*^	Langtan NP, Nepal	Oct '00 - May '01	several groups	27.0	28.5		28.5	16.0		11, S
*M. assamensis*	Phu Khieo WS, Thailand	2007-'08, 2010-'11	49-53 (AS)^e^	24.7	4.9	27.2	32.0	11.1		12, F
*M. assamensis*	Jokai forest, Assam, India	Jun '97 - May '98	31	25.0	40		22	13.0		13, S
*M. nigra*	Tangkoko, Sulawesi	Jan '93 - Jun '94	42-50 (Mal.)	25.7	13.8	24.8	12.6	23.1		14, S
*M. nigra*	Tangkoko, Sulawesi	Jan '93 - Jun '94	57-61 (Dua)	23.5	15.2	20.8	17	23.5		14, S
*M. nigra*	Tangkoko, Sulawesi	Jan '93 - Jun '94	76-97 (Ram.)	18.3	9	25.1	28.9	18.7		14, S
*M. nigra*	Tangkoko, Sulawesi	Jul '06 - Jun '07	60 (Ram. I)	12.4	54.1		16.9	16.1	0.4	15, S
*M. fascicularis*	Kuala Lompat, W-Malaysia	Jul '74 - Jan '76	23	20.0	35.0		34.0	12.0	0.5	16, S
*M. fascicularis*	Ketambe, Sumatra	Mar '10 - Apr '11	28 (KA)^f^	6.1	3.8	38.7	48.2	5.9		17, F
*M. fascicularis*	Ketambe, Sumatra	Mar '10 - Apr '11	52 (C)^f^	3.8	2.3	36.3	47.2	10.7		17, F
*M. fascicularis*	Ketambe, Sumatra	Mar '10 - Apr '11	35 (KB)^f^	3.6	3.4	34.8	49.3	10.0		17, F
*M. nigrescens*	Dumoga-Bone NP, N-Sulawesi	Apr '89 - Jun '90	13.9	19.7	10.1		47.6	22.7		18, S
*M. munzala*	Arunachal Pradesh, NE-India	Jul - Aug '05	13 (Ro.), 22 (Br.)	19.0	29.0		36.0	16.0		19, O
*M. cyclopis*	Mt. Longevity, Taiwan	Aug '03 - Jul '04	16-63 (Aa, C, E, F)	16.0	8.2	28.1	17.1	30.2		20, U
*M. cyclopis*	Yushan NP, Taiwan	Mar '87 - Oct '88	7.8	9.5	52.8		30.9	x	6.7	21, S
M. radiata *	Bandipur-Mundumalai, India	1 year	15	7.0	37.0		21.0	30.0	5.0	22, F
**Temperate climate**										
*M. mulatta*	India	1981 - '84	31.5 (70 groups)	26.2	40.1		27.7	4.7	1.3	23, U
*M. mulatta* ^*b*^	Kathmandu, Nepal	1974 - '75	x	25.0	27.0		8.0	21.0	21.0	24, S
*M. mulatta*	N-India	1981 - '86	43-70 (3 groups)	24.1	33.6		35.1	5.6	1.5	25, S
*M. mulatta*	Murree Hills, NW-Pakistan	1978 - '79	23-25 (Kong)	11.0	45.0		34.0	10.0	x	26, F
*M. fuscata yakui*	Yakushima, Japan	Jan '90 - May '92	5-19 (P)	23.0	32.7		22.6	18.9	2.8	27, S
*M. fuscata yakui*	Yakushima, Japan	Aug - Dec '76	47	22.8	23.5		22.1	31.6		28, S
*M. fuscata yakui*	Yakushima, Japan	1976, 1989-'92	5-19 (P, T, Ko)	22.6	30.8		22.1	20.7	3.7	29, S
*M. fuscata yakui*	Yakushima, Japan	Apr '00 - Mar '01	24 (HR)	16.0	38.0		32.0	14.0		30, F
*M. f. fuscata*	Kinkazan Island, Japan	1984-'87, '91-'92	20-51 (A)	16.8	53.9		17.6	11.5	0.3	29, S
*M. f. fuscata* ^*d*^	Kinkazan Island, Japan	Sep - Dec '89	38 (A)	13.5	60.3		6.4	14.6	1.9	31, U
*M. sylvanus*	Akfadou, Algeria	Feb '83 - Mar '85	33-41	22.3	3.9	23.8	40.0	10.0		32, S
*M. sylvanus*	Ain Kahla, Marocco	1968 - '69	25 (6 groups)	21.8	50.1		16.7	10.8	0.6	33, S
*M. sylvanus*	Djurdjura, Algeria	Feb '83 - Mar '85	38-47	20.0	6.2	25.4	36.9	11.5		32, S

Obs.M. = Observation method (S = scan sampling, F = focal animal sampling, O = other method, U = unknown); NP = National Park, WS = Wildlife Sanctuary; ^a^ group was living in disturbed forest, ^b^ group was feeding to some extent on human food, ^c^ values averaged from both years, ^d^ values averaged from different age-sex categories, ^e^ data are only from adult females (12 in 2007/08; 15 in 2011/12), ^f^ data are only from the 2 adult males, * values were estimated from figure

Sources and habitat type: 1) Caldecott (1986a), tropical broadleaf evergreen forest surrounded by oil palm plantations; 2) this study, tropical lowland evergreen broadleaf rainforest; 3) Singh et al (2000), evergreen moist broadleaf forest; 4) Menon & Poirier (1996), disturbed forest fragment; 5) Kurup & Kumar (1993), undisturbed wet evergreen forest; 6) Riley (2007), lowland and hill forest, Ch group minimally altered, Anca group heavily altered with agricultural and agroforestry areas; 7) Pombo et al (2004), smaller group in undisturbed forest, larger group in disturbed forest; 8) Albert (2012), seasonal wet evergreen forest, close to human settlement; 9) Choudhury (2008), tropical wet evergreen forest, deciduous plantations; 10) Aggimarangsee (1992) in Chalise (1999); 11) Chalise (2003), at Makalu-Barun National Park steep slopes with patchy forest, for Langtan National Park habitat not mentioned; 12) M. Heesen (pers. comm.), dry evergreen forest; 13) Sarkar et al (2012), semi-evergreen forest; 14) O’Brien & Kinnaird (O'Brien and Kinnaird 1997), different percentage of primary forest for the different groups (Mal.: 15%, Dua: 20%, Ram.: 4%), rest is secondary and burned forest; 15) Giyarto (2010), mainly primary forest; 16) Aldrich-Blake (1980), tropical lowland evergreen rainforest; 17) C. Girard-Buttoz (pers. comm.), tropical lowland evergreen rainforest; 18) Kohlhaas (1993), primary lowland rainforest, with some patches of secondary growth and grasses; 19) Kumar et al (2006), subtropical broadleaf evergreen forest, secondary scrub and agricultural fields; 20) Wang (2004); 21) Lu et al (1991), mainly primary broadleaf forest; 22) Singh & Vinanthe (1990), dry decidious forest; 23) Seth & Seth (1986), deciduous forest; 24) Teas et al (1980), open and wooded parklands, small tracts of forest, temple grouds; 25) Chopra et al (1992), forest; 26) Goldstein & Richard (1989) and Goldstein (1984), temperate mixed coniferous deciduous forest with disturbed areas; 27) Agetsuma (1995b), warm temperate broadleaf forest; 28) Maruhashi (1981), warm temperate broadleaf forest; 29) Agetsuma & Nakagawa (1998), Yakushima: warm temperate broadleaf forest, Kinkazan: mixed forest of deciduous and coniferous trees; 30) Hanya (2004b), coniferous forest; 31) Hashimoto (1991), deciduous broadleaf forest; 32) Ménard & Vallet (1997), Akfadou: temperate deciduous oak forest, Djurdjura: temperate evergreen cedar-oak forest; 33) Deag (1985), temperate cedar forest.

### Diet

Across the study period, the diet of Siberut macaques was on average composed of 75.7% fruit (72.8% ripe fruit, 20.2% half-ripe fruit, 4.3% unripe fruit, 2.7% fruit of unknown ripeness), 11.9% arthropods (mainly ants, termites, spiders), 4.5% mushrooms, 4.4% leaves (59.7% young leaves, 13.4% young leave petioles, 25.4% leaves of unknown age, 1.5% mature leaves), 2.6% pith, 0.6% sap, 0.2% shoots and 0.2% flowers. Compared to other macaques, the degree of frugivory in Siberut macaques was high (third highest among the 14 macaque species examined) and the percentage of leaves eaten low (fifth lowest of the 14 macaque species, Table 4). According to local people, Siberut macaques also occasionally catch and consume crabs and shrimp from the rivers, but this was only observed once. In contrast to other macaque species, Siberut macaques were never observed to prey on bird eggs, birds, squirrels or other small mammals. Although the majority of the diet was comprised of fruit, the proportion of fruit varied largely from 43.2% (Dec. 2010) to 96.1% (Apr. 2010, Figure 2). With decreasing proportion of fruit eaten per month, the time spent feeding on arthropods, pith and leaves increased significantly (Spearman rank correlations: fruit vs. arthropods: r_s_ = -0.97, p<0.01, fruit vs. pith: r_s_ = -0.86, p<0.01, fruit vs. leaves: r_s_ = -0.59, p<0.05, for all N=12 months). The correlation between fruit and mushrooms was not significant. The increase in pith eating was mainly due to adult males, since all males fed on pith whereas only 2 of the 6 adult females did. Pith was the only food item which was significantly different in the diet of males and females (Mann-Whitney-U: W=18, p=0.02). Observations suggested that only males were strong enough to break the palm trunks open to get access to the pith, whereas females were only observed feeding pith after they found a trunk already opened. In sum, when the abundance of fruit decreased, they used arthropods and (young) leaves as fallback foods (for annual availability of leaves and fruit see Erb et al. 2012a).

**Table 4:** **Diet (as percent of feeding time) of free-ranging and unprovisioned macaque groups of different species, sorted by decreasing percentage of fruit eaten and by species**

Species	Fruits (Pods)	Flowers	Seeds	Leaves	Buds	Shoots	Herbs	Stem	Pith	Bark	Roots	Sap/Resin	Fungi	Lichens	Invertebrates	Vertebrates	Other	Source
**Tropical/subtropical climate**																		
*M. tonkeana*	85.8	0.8		4.2		3.1		0					0.3		5.6		0.4	1
*M. tonkeana*	78.1	0.8		2.9		1.8		0.3					1		14.6		0.4	1
*M. nigrescens*	85.1	y		3.5		y		y							8.9		2.5	2
***M. siberu***	75.7	0.2		4.4		0.2			2.6			0.6	4.5		11.9			3
*M. n. nemestrina*	74.6	1.1		7.0	3.0			1.9							12.2	0.4		4
*M. fascicularis*	66.7^a^	8.9	y	17.2											4.1		3.2	5
*M. fascicularis*	63.7	8.8		24.0											4.4			6
*M. fascicularis*	52.4	5.4		16.1							2.9				23.3			7
*M. fascicularis*	44.9	6.5		8.4				6.3		7.9			6.6		11.3		8.0	8
*M. fascicularis*	30.0	4.1		10.5				4.4		30.2			1.8		15.5		3.4	8
*M. fascicularis*	25.0	1	45.0^b^	9.0				13.0^c^							5.0		2	9
*M. fascicularis*	15.1	6.4		5.5				4.1		44.9			2.7		19.5		1.8	8
*M. fascicularis*	4.0			41.0					6.0^d^						46.0			10
*M. nigra*	66.0	y	y	2.5 (+ y)		y	y		y				y		31.5			11
*M. nigra*	61.5	0.1		8.1 (+ y)	y			y					2.5		27.6	0.2	0.1	12
*M. nigra*	56.9	0.1		9.0 (+ y)	y			y					1.1		31.7	0.1	0.8	12
*M. (n.) leonina*	65.9	2.1	6.1	7.7^e^	y	1.5		y			3.5^f^				11.7		1.5	13
*M. silenus*	59.5			2.0											18.0		20.6	14
*M. cyclopis*	53.8	7.3	0.0	14.9		2.4		11.8							9.8			15
*M. cyclopis*	42.2	10.3		26.2	9.1			11.8			0.03							16
*M. assamensis*	30.7	2.0	28.2	12.4		0.7				0.3	0.3		0.1		20.6	0.1	4.5	17
*M. assamensis*	22.9	31.4		45.7														18
*M. assamensis*	11.0	7.0		52.0	30.0										2.0			19
*M. munzala*	10.3	3.25	0	40.2					41.4								4.85	20
**Temperate climate**																		
*M. fuscata yakui*	30.2	5.6	13.2	35.1^g^		y			y	y		y	4.6		10.3		1.2	21
*M. fuscata yakui*	28.6	4.9	28.2	22.4											8.9		7.0	22
*M. fuscata yakui*	13.0	15.0	4.0	41.0									14.0		1.0		11.0	23
*M. f. fuscata*	10.2	3.3	43.6	14.4	2.9		15.1			5.0			2.3		2.1		1.3	24
*M. mulatta*	8.5	3.7		84.4 (+ y)			y	y			2.2	1.1						25
*M. sylvanus*	0.8	3.5	32.2	8.8			18.5				6.9		4.1	14.2	10.5		0.5	26
*M. sylvanus*	4.3	1.6	26.7	13.0			35.1				7.7		1.5	1.9	5.6		2.6	26

For more details of the studies see Table 5.

Leaves includes leaves of trees, shrubs and lianas of different stages of maturity or fallen leaves, leaf petioles and palm fronds. The category flowers also includes flower buds. The category buds usually means leaf buds, and in case it was not specified in the literature whether flower or leaf buds were meant, the values were included in this category as well.

y = yes this food item was eaten but exact value not given by the author, and was either summarized with another food item (mentioned there) or given in the category other, (+y) = this value includes all other food items for which y is entered, ^a^ includes seeds, ^b^ includes pods, ^c^ includes bracts, ^d^ includes nectar, ^e^ includes buds, ^f^ includes stem, ^g^ includes shoots.

Sources: 1) Riley (2007), 2) Kohlhaas (1993), 3) this study, 4) Caldecott (1986a) and Caldecott (1986b), 5) Yeager (1996), 6) MacKinnon & MacKinnon (1980), 7) Aldrich-Blake (1980), 8) C. Girard-Buttoz (pers. comm.), 9) Sussman & Tattersall (1981), 10) Khan & Wahab (1983) in Ahsan (1994), 11) O’Brian & Kinnaird (1997), 12) Giyarto (2010), 13) Choudhury (2008), 14) Singh et al (2000), 15) Su & Lee (2001), 16) Wang (2004), 17) M. Heesen (pers. comm.), 18) Ahsan (1994), 19) Srivastava (1999), 20) Mendiratta et al (2009), 21) Hill (1997), 22) Agetsuma (1995a), 23) Hanya (2004a), 24) Agetsuma & Nakagawa (1998), 25) Goldstein & Richard (1989) and Goldstein (1984), 26) Ménard (2004).

**Table 5:** **Details of the studies mentioned in Table** 4

Species	Study site	Habitat type	Study period	Group size (name)	Source
**Tropical/subtropical climate**					
*M. tonkeana*	Lore Lindu NP, Sulawesi	Lowland and hill forest, minimally altered	Jan '03 - Apr '04	26-28 (Ch)	1
*M. tonkeana*	Lore Lindu NP, Sulawesi	Lowland and hill forest, heavily altered	Jan '03 - Apr '04	6-9 (Anca)	1
*M. nigrescens*	Dumoga-Bone NP, Sulawesi	Primary lowland rainforest, secondary growth	Apr '89 - Jun '90	13.9	2
***M. siberu***	Siberut island, Sumatra	Tropical lowland evergreen broadleaf rainforest	Mar '10 - Mar '11	29	3
*M. n. nemestrina*	Lima Belas, W-Malaysia	Trop. broadleaf evergreen forest, plantations around	Jan '80 - May '81	50	4
*M. fascicularis*	Tanjung Puting, Kalimantan	Freshwater peat swamp forest	Jan - Dec '85	(several)	5
*M. fascicularis*	Kuala Lompat, W-Malaysia	Tropical lowland evergreen rainforest	Jan - Jul '73	17	6
*M. fascicularis*	Kuala Lompat, W-Malaysia	Tropical lowland evergreen rainforest	Jul '74 - Jan '76	23	7
*M. fascicularis*	Ketambe, Sumatra	Tropical lowland evergreen rainforest	Mar '10 - Apr '11	52 (C)*	8
*M. fascicularis*	Ketambe, Sumatra	Tropical lowland evergreen rainforest	Mar '10 - Apr '11	35 (KB)*	8
*M. fascicularis*	Mauritius	Degraded savanna	Jun - Jul '77	67	9
*M. fascicularis*	Ketambe, Sumatra	Tropical lowland evergreen rainforest	Mar '10 - Apr '11	28 (KA)*	8
*M. fascicularis*	Naaf river belt, Bangladesh	x	x	20	10
*M. nigra*	Tangkoko, Sulawesi	Lowland rainforest	Jan '93 - Jun '94	42-97	11
*M. nigra*	Tangkoko, Sulawesi	Lowland rainforest	Jul ’06 – Jun ‘07	60 (Ram. I)	12
*M. nigra*	Tangkoko, Sulawesi	Lowland rainforest	Jul ’06 – Jun ‘07	58 (Ram. II)	12
*M. (n.) leonina*	Bherjan, E-India	Tropical wet evergreen forest, deciduous plantations	1992-1994, 2004	20-23 (2 groups)	13
*M. silenus*	Western Ghats, S-India	Evergreen moist broadleaf forest	Aug '94 - Mar '96	23	14
*M. cyclopis*	Jentse, NE-Taiwan	Secondary broadleaf evergreen forest, plantations	Oct '91 - Jun '92	≤31 (≥ 6 groups)	15
*M. cyclopis*	Mt. Longevity, Taiwan	x	Aug '03 - Jul '04	16-63 (Aa, C, E, F)	16
*M. assamensis*	Phu Khieo WS, Thailand	Dry evergreen forest	2007-'08, 2010-'11	49-53 (AS)	17
*M. assamensis*	Bangladesh	x	1979 - 1981	18	18
*M. assamensis*	Jokai RF, Assam, India	x	x	x	19
*M. munzala*	Arunachal Pradesh, India	Subtropical broadleaf forest, disturbed open forest	Dec '05 - May '06	24	20
**Temperate climate**					
*M. fuscata yakui*	Yakushima, Japan	Warm temperate broadleaf forest (coastal forest)	Dec '87 - May '89	15-17 (P)	21
*M. fuscata yakui*	Yakushima, Japan	Warm temperate broadleaf forest	Jan '90 - Apr '92	5-19 (P)	22
*M. fuscata yakui*	Yakushima, Japan	Cold temperate coniferous forest	Apr '00 - Mar '01	24-27	23
*M. f. fuscata*	Kinkazan Island, Japan	Mixed forest of deciduous and coniferous trees	Nov '84 - Aug '92	20-51 (A)	24
*M. mulatta*	Murree hills, NW-Pakistan	Mixed coniferous deciduous forest, disturbed areas	1978-'79	23-25 (Kong)	25
*M. sylvanus*	Akfadou, Algeria	Temperate deciduous oak forest	Feb '83 - Mar '85	33-47	26
*M. sylvanus*	Djurdjura, Algeria	Temperate evergreen cedar-oak forest	Feb '83 - Mar '85	38-73	26

NP = National Park, RF = Reserve Forest, WS = Wildlife Sanctuary, * data are only from the 2 adult males, Sources: see footnotes of Table 4.

Dietary diversity, measured by the Shannon-Wiener index H’, was low in months when the proportion of fruit in the diet exceeded 80% (average H’: 1.1) and high in months when fruit only made up a smaller part of the diet (average H’: 2.1). Overall average dietary diversity was 1.6. The monthly dietary diversity index was negatively correlated with the monthly feeding time (Spearman rank correlation: r_s_ = -0.82, N= 12, p=0.02), indicating that the monkeys need to invest more time in feeding when the diversity of food items is low.

### Comparison of Siberut’s primates

Of all four sympatric primate species on Siberut, Siberut macaques have the largest group size and by far the largest home range size (see Table 1 for data and references). The size of home range increases with the percentage of fruit in the diet across species, with Siberut macaques being the most frugivorous species. At similar proportions of fruit and leaves in the diet, Kloss gibbons spend twice as much time feeding on arthropods, and Siberut macaques included more other food items instead, thus having a broader diet (Table 1). The amount of frugivory also seems to be related to the daily travel distance, with both folivorous colobine species (*Presbytis potenziani* and *Simias concolor*) traveling the shortest distances, Kloss gibbons being intermediate and Siberut macaques having 3-4 times the travel distances of the two colobine species; the same pattern emerges for travel time (Table 1). For the forest strata use we find a niche differentiation. Whereas Siberut macaques mainly used the lower strata (0-10 m) of the forest, the sympatric colobine species mainly stayed within heights of 11 to 20 m (Table 1). For Kloss gibbons, no data are available for the same study site but data collected in Central Siberut indicate that the gibbons spent 94% of their time in the middle and upper canopy (Whitten 1982c).

### Habitat analysis

Within the 3 ha of forest sampled within the home range, a total of 1,807 individuals of trees, palm trees, rattan and lianas, belonging to 167 species, 107 genera and 46 families were recorded. 107 individuals (5.9%; 5 lianas and 102 trees) could not be identified at the family level. From the remaining individuals, 83% could be determined to species level, the rest to genus level. Trees were the dominant growth form, with 40 families and 133 species. The liana flora comprised 12 families and 19 species. This natural and undisturbed forest had 3 species of palm trees (*Arenga obtusifolia*, *Oncosperma horridum*, *Pinanga* sp.) and 10 species of rattan (*Calamus*: 6, *Korthalsia*: 3, *Plectocomia*: 1). We found two strangler species (*Ficus annulata*, *Ficus sp.*, Moraceae). The estimated potential species richness for all categories (trees, palm trees, lianas, rattan and strangler) ranged between 186 and 225, with a mean of 200 species for the different estimators (ACE: 188, ICE: 203, Chao 1: 193, Chao 2: 200, Jack 1: 207, Jack 2: 225, Bootstrap: 186; MMMeans (1^st^ run): 200). Estimated tree species richness ranged between 150 and 189, with a mean of 166 species for the different estimators (ACE: 154, ICE: 169, Chao 1: 164, Chao 2: 177, Jack 1: 170, Jack 2: 189, Bootstrap: 150: MMMeans (1^st^ run): 160). For lianas, a mean of 22 species was estimated, with a range of 20 to 24 (ACE: 21, ICE: 22, Chao 1 & Chao 2: 20, Jack 1 & Jack 2: 23, Bootstrap: 21: MMMeans (1^st^ run): 24). For palm trees, rattan and strangler, the estimated species richness was the same as the observed one. In sum, total species richness observed was 167 and expected was 200, which is mainly due to the tree community which was undersampled. These estimates were in line with field observations on species occurrence outside the plots.

Looking at species diversity, which combines the information of species richness and relative abundance, we found a Simpson’s diversity index of 0.97 for all categories together (trees, palm trees, liana, rattan, strangler), or 0.98 when only considering trees. As this index ranges between 0 and 1, with 1 being the highest diversity, this indicates a very high species diversity of the studied forest habitat. Similarly, the Shannon-Wiener diversity index was also high, with 4.30 for all categories, and 4.32 for trees alone. The 12 botanical plots studied showed a mean similarity in terms of species composition ranging from 0.32 to 0.63, depending on the index (incidence based indices: Jaccard: 0.32, Sørensen: 0.49, but both are usually biased downward when species richness is large; abundance based indices: Morisita-Horn: 0.60, Bray-Curtis: 0.43, Chao-Jaccard: 0.47, Chao-Sørensen: 0.63), resulting in a mean of 0.49, which is a medium similarity within the indices possible range between 0 and 1.

The average total density of all recorded individuals per area was 602.3 per ha (±118.1 SD) (trees: 402.3 individuals/ha (±86.8), palm trees: 73.0 individuals/ha (±24.8), lianas: 41.0 individuals/ha (±19.9), rattan: 83.3 individuals/ha (±52.7), strangler: 2.7 individuals/ha (±3.1)).

The height distribution of the forest showed tree heights ranging from 2 m (*Chionanthus glomerata*, Oleaceae) up to maximum 52 m (*Sloanea javanica*, Elaeocarpaceae). Other high trees taller than 40 m were *Nauclea* sp. (Rubiaceae), *Scorodocarpus borneensis* (Olacaceae), *Syzy-gium palembanicum* (Myrtaceae) and *Palaquium obova-tum* (Sapotaceae). Most of the trees (76%) fall into the three height categories from 6 to 20 m (Figure 5). When considering trees and palm trees together (as Siberut macaques often used palm trees as food resource), these three categories account for 78% (Figure 5). The category with the highest proportion of trees, 11-15 m, accounted for one third of all trees (32.6% for trees, 33.1% for trees & palm trees). Only a small proportion of trees was higher than 30 m (trees: 4.1%, trees & palm trees: 3.6%). The distribution of time the macaques spent in different height classes during their normal, daylight activities was different from the abundance of different height classes in the forest (&Chi;^2^ = 1841.5, df = 8, p<0.001, Figure 5) The macaques spent 51% of their time at less than 5 m height, and only 30% of their time between 6 and 20 m, the three categories which comprise the majority of the forest trees.

**Figure 9:**
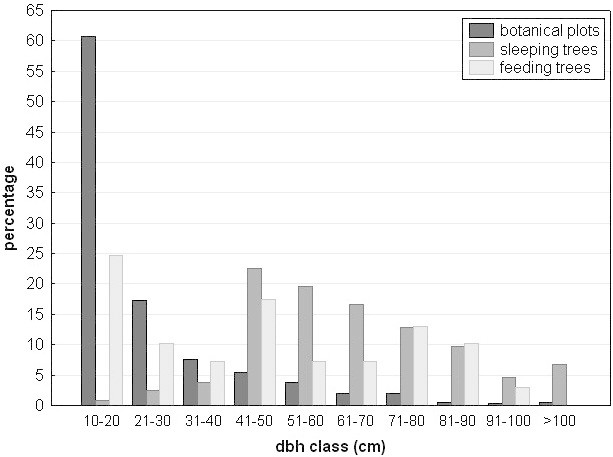
**Distribution of dbh classes (in cm) of trees (without palm trees) of all botanical plots, of sleeping trees and of feeding trees.**

The diameter distribution of trees showed an average of 99 cm dbh in all plots together, which is mainly driven by the large trees characteristic for Plot A, the only plot on the top of a hill. After excluding this plot, the average dbh of trees decreased to 38 cm. The largest trees >100 cm dbh belonged to the family Dipterocar-paceae (*Dipterocarpus elongates*, *Shorea ovalis*, *Shorea pauciflora*) in Plot A, and to the family Euphorbiaceae (*Endospermum malaccense*) and Moraceae (*Artocarpus maingayi*, *Ficus* sp.) in 2 plots on the dry level ground. The distribution of trees in the botanical plots per dbh class followed a negative exponential distribution, with trees of smaller diameters being the most abundant, with a gradual decrease with increasing diameter (Figure 9). The sleeping trees used by the study group showed a very different distribution from the actual forest tree distribution (Figure 9; &Chi;^2^ = 647.8, df = 9, p<0.001). The majority of sleeping trees (85%) had a dbh between 40 and 90 cm. Nearly 7% of sleeping trees were larger than 100 cm dbh, indicating that monkeys favored large trees as sleeping trees. As large trees occur at low abundance they are an important requirement for a suitable habitat of Siberut macaques.

The distribution of feeding tree sizes was more even (Figure 9). A large proportion of feeding trees were small trees between 10 and 20 cm dbh (24.6%), but there was also a high percentage of feeding trees larger than 50 cm dbh (40.6%). The distribution of feeding trees used by the group was significantly different from the distribution of the same tree species in the botanical plots (&Chi;^2^ = 74.3, df = 9, p<0.001), thus indicating that large feeding trees are a critical key component for the survival of Siberut macaques.

The tree flora was clearly dominated by the family Euphorbiaceae, both in terms of number of individuals (156) and species richness (21 species). For Siberut macaques, this family was important because it included the most favorite species of sleeping trees (*Endospermum malaccense*). As fruit resource, Euphorbiaceae appeared to be less important, which may be due to the irregularity of fruit production. Five of the seven species used by the macaques were fruiting in one year (habituation period), but not in the other (data collection period). The second most important tree family in terms of number of individuals (119) was Myristicaceae. It had a high importance as fruit resource for Siberut macaques (*Knema* sp.), but not as much as sleeping trees. Other important families in the forest based on number of individuals were Dipterocarpaceae (78), Sapotaceae (68) and Myrtaceae (63). In terms of species richness however, Euphorbiaceae, the family with the highest species richness, was followed by Lauraceae (9), Myristicaceae (8), Annonanceae (7) and Rubiaceae (7).

Considering all studied plant categories in the botanical plots and not only trees, the species with the highest abundance was a rattan species, *Korthalsia echinometra* (Palmae), reaching an abundance of 158 individuals in the 3 ha of forest examined, followed by the palm tree *Oncosperma horridum* (157 individuals). The most abundant tree species *Vatica pallida* (Dipterocarpaceae) was about three times rarer in number (61 individuals). Rattan and palm trees were very important for Siberut macaques because they provided fruit for a longer time period. This can be seen from the number of months each species was recorded as fruit resource during scan observations over a period of 1 year. In average, palm trees provided fruit during 6.0 months (N= 4 species), rattan during 3.3 months (N= 8), whereas lianas and trees only during 2.1 or 2.0 months respectively (N= 7; N= 17).

The importance of palms for the forest and their dominance can also be seen from the basal area per family: Palmae (or Arecaceae) was the top-ranking family in terms of basal area (4.86 m^2^/ha), followed by Dipterocarpaceae (2.98 m^2^/ha), Euphorbiaceae (2.68 m^2^/ha), Myristicaceae (2.51 m^2^/ha), Moraceae (2.46 m^2^/ha), Myrtaceae (2.27 m^2^/ha), and Sapotaceae (2.09 m^2^/ha). All other families had a basal area less than 2 m^2^/ha. Total basal area of trees, palm trees, lianas, rattan and strangler combined was 33.68 m^2^/ha, that of trees alone 28.46 m^2^/ha.

## Discussion and conclusions

### Interspecies comparisons: the genus Macaca

The present study provides first comprehensive data on the ecology of a new representative within the ecologically very diverse macaque genus. The study group comprised 29 individuals, which is twice the size suggested by the preliminary observations of Whitten & Whitten (1982). Our second semi-habituated group consisted of at least 13 individuals, which is probably an underestimate as the number of adult males (3) which were frequently seen and vocalized was the same as in our much larger study group. All these observations suggest that group size for Siberut macaques falls within the range reported for *M. silenus* (9-31 individuals; 9 groups: Kumara & Singh (2004)), *M. sinica* (5-47 individuals; 20 groups Dittus (1988)) and *M. radiata* (16-44 individuals; 12 groups: Sugiyama (1971)).

(CaldecottOur comparative data sets revealed that Siberut macaques are ecologically most similar to pigtail macaques, *M. nemestrina*^1986^a;Caldecott et al. 1997), their sister taxa according to genetic analyses (Ziegler et al. 2007). Siberut macaques are semi-terrestrial, traveling large distances per day relative to their group size, spending a very high percentage of their daily activities on traveling and using a mainly frugivorous diet. In the following, we discuss these traits in more detail.

The Siberut macaques studied here spent a very large amount of their daily activities on traveling, ranking second among all 15 macaque species examined. These differences in traveling time appear to be true differences, as they cannot be attributed to different sampling methods (69% scan sampling, 21% other, 10% unknown observation method; Table 3) or different definitions, because even when taking both traveling and foraging time together, Siberut macaques are still different from other macaque species. Siberut macaques also traveled longer distances per day (highest value for their group size, but similar to *M. nemestrina*). The high amount and distance traveled may be linked to the degree of terrestriality. For pigtail macaques, ground foraging and traveling is an adaptation to the habitat of Sundaic dipterocarp forest with scarce food resources which are patchy and slow to renew (Caldecott 1986a). Ground traveling allows fast traveling between widely dispersed fruit resources, and additionally allows the exploitation of food resources on the ground (Caldecott 1986a;Caldecott et al. 1997). Similarly, Siberut macaques inhabit dipterocarp and mixed forest where food seemed to be dispersed and often in small patches, for example the various different rattan fruit or Aren (*Arenga obtusifolia*) fruit which were common fruit resources throughout the year (CR, unpublished data). Similar to pigtail macaques, Siberut macaques mainly used the ground for traveling and to some extent for foraging. They were usually searching for insects or spiders and mushrooms under old foliage or on fall-down trees, and were occasionally picking young leaves or leaf petioles of herbs from the ground vegetation (e.g. from *Curculigo latifolia*, Hypoxidaceae), while searching for fruiting trees within their home range. As adaptation to those scattered and often cryptic resources, they would often forage alone or together with only a few other individuals in close vicinity, with sometimes large group spreads of ~200 m, or in rare cases of over 400 m. A similar pattern was reported for pigtail macaques (Caldecott 1986a;Caldecott et al. 1997). Another explanation for the high amount of traveling in Siberut macaques could be the lower percentage of trees bearing fruit (max. 5% in Central Siberut (median 3.5%) compared with max. 16% (median 4%) in Malay Peninsula (Whitten 1980)).

Comparison of the diets of macaques revealed that Siberut macaques were mainly frugivorous, which is similar to *M. nemestrina*, *M. nigrescens* and *M. tonkeana* (Table 4). Ménard (2004) suggested that the most frugivorous macaque species also spend the most time moving. We tested this using the comparative data sets from Tables 3 and 4 and by only including those groups and species for which both the time spent feeding on fruit and the time spent moving/ traveling were available for the same study site or group, we avoided potential confounding effects of habitat differences. There was a positive significant correlation (Spearman rank: r_s_ = 0.53, N=20, p<0.02), i.e. the more frugivorous a species (or group) is, the more time of its daily activities it has to spend traveling, which could partly explain the large amount of time spent traveling in Siberut macaques.

Figs (Ficus spp.) are a common food source for many primates (Shanahan et al. 2001) and can make up a large percentage of the fruit diet of macaques ( (Ungar20-40% for *M. fascicularis*^1995^;Kinnaird and O’Brien 2005), 44% for *M. nigra* (Kinnaird and O’Brien 2005(Kohlhaas), 47% for *M. nigrescens*^1993^); see also Riley (2007) for the importance for *M. tonkeana*). For Siberut macaques however, figs only accounted on average for 6.9% of the total amount of known fruit eaten per month. The abundance of figs (≥ 10 cm dbh) on Siberut was relatively low, with a density of 2.7 figs/ha, similar to the figure for South Sumatra (1.4 figs/ha, Kinnaird & O’Brian (Kinnaird and O'Brien 2005)), but very different to Sulawesi (11.8 figs/ha in the habitat of *M. nigra* in North Sulawesi (Kinnaird and O'Brien 2005), 33.2 figs/ha in the habitat of *M. tonkeana* in Central Sulawesi (Riley 2007)). As figs are not so abundant in the habitat of Siberut macaques, they are of lesser importance than, for example, rattan or palm fruit (rattan fruit: 8.5%, palm tree fruit: 22.3% of the monthly fruit diet). A high degree of frugivory and thus dependence on fruiting trees within the forest has important conservation consequences for *Macaca siberu*, which will be discussed below.

In addition, as island biogeography theory would predict, we may expect a possible niche shift or niche expansion in Siberut macaques, since longtail macaques which are the main competitors of pigtail macaques on the mainland Sumatra (Crockett and Wilson 1980), are completely absent in Mentawai. However, as the activity budget and diet of Siberut macaques and pigtail macaques are very similar, there is no indication for a behavioral or dietary niche differentiation. For forest strata use, no detailed data are available for pigtail macaques, but judging from the degree of terrestriality, there is also no indication for a broadened niche.

### Interspecies comparisons: Siberut’s primates

Of all primate species on Siberut, Siberut macaques have the largest group size and the largest home range requirements (see also Whitten and Whitten 1982). Although Kloss gibbons and Siberut macaques are both mainly frugivorous, Siberut macaques have a broader diet (i.e. also feed on herbs, mushrooms, sap and resin, pith etc) and dietary overlap between the two species seems low (only 25.6%, i.e. 10 out of 39 fruit species recorded for Kloss gibbons (Whitten 1982a) were also used by Siberut macaques, but different species may fruit in different years). Our present findings showed that Siberut macaques consumed a remarkably small amount of leaves for a rainforest macaque species (Table 4) and in this respect they resemble Kloss gibbons which include a much lower proportion of leaves in their diet compared to Malaysian gibbon species (Whitten 1982a). One possible explanation may be that due to the high annual rainfall and a very nutrient poor soil on Siberut, competition between trees is especially high resulting in a high concentration of secondary compounds in (tree) leaves and thus in a decreased digestability for mainly frugivorous primates, leading to a decreased choice of leaves as food resource compared to other primate species (Whitten 1980;Whitten 1982a). Quantitative data to test this, however, are missing. Siberut macaques still fed on more leaves than Kloss gibbons, probably because they also used young herbaceous leaves from the understory vegetation, as they frequently foraged and travelled on the ground.

### Forest comparison

The behavior of every animal is closely linked and determined by its habitat (Krebs and Davies 1997). Differences in the behavior of Siberut macaques compared to other macaque species could be attributed to differences in the forest habitat. To investigate this possibility, we compared species, family richness and other important forest characteristics of the habitat of Siberut macaques with other forests in the same phytogeographical region of Malesia.

In our study, we recorded a tree species richness of 133, which is lower than in North-Sumatra (184 species, Kartawinata et al. 2004), and thus in line with island biogeography theory (MacArthur and Wilson 1967;Simberloff 1974), as islands usually have an impoverished flora. This impoverishment, however, was only seen at the species level, not at the family level, as we recorded a similar number of tree families as in North-Sumatra (40 families in this study; 41 in North Sumatra: Kartawinata et al. 2004). The basal area of trees in our study site was very similar to that recorded in Central Siberut (28.5 m^2^/ha of trees ≥10 cm dbh in this study; 27.7 m^2^/ha of trees ≥15 cm dbh in Central Siberut, Whitten (1982d)), indicating that our botanical plots are representative for the forest of Siberut Island. In North-Sumatra, however, basal area is much higher (40.6 m^2^/ha of trees ≥10 cm dbh, Kartawinata et al (2004). The three most species rich families were Euphorbiaceae, Lauraceae and Myristicaceae, with Euphorbiaceae and Lauraceae also being among the three most species rich families in another study at the same site in Siberut (Hadi et al. 2009b), as well as in West and East Malaysia (Kochummen et al. 1990;Lee et al. 2002). The tree families with the highest richness of individuals were Euphorbiaceae, Myristicaceae and Dipterocarpaceae, which is the same as in Central Siberut but in a different order (Dipterocarpaceae, Myristicaceae, Euphorbiaceae: Whitten 1982d), and Euphorbiaceae and Dipterocarpaceae were also the two top-ranking families in terms of tree richness in mainland Sumatra, West and East Malaysia (Kochummen et al. 1990;Laumonier 1997;Lee et al. 2002).

Rattan, which are centered in their distribution on the Sunda Shelf (Whitmore 1984), were an important feature of the studied forest, reaching densities of 83.3 individuals (apparent genets) per hectare. Although this density is much lower than in West Malaysia (115 clumps/ ha, Abdul Hamid and Suratman 2010) or Sulawesi (314 mature genets/ha, Siebert 2005), rattan diversity (10 species in this study) was higher than in East Malaysia (6 species, Putz and Chai 1987) and similarly high in West Malaysia (11 species, Abdul Hamid and Suratman 2010). Apart from rattan, palm trees were a common element in the forest, as already noticed for Central Siberut before (Whitten 1982d), reaching relatively high densities (*Oncosperma horridum*: 52.3 palms ≥10 cm dbh/ha, *Arenga obtusifolia*: 17.3 palms ≥5 cm dbh/ha or 9.7 palms ≥10 cm dbh/ha). They are no indicator of disturbance, as they are common in primary forests (Laumonier 1997). Densities of other species which are more light demanding and thus common in secondary forests (Kochummen et al. 1990) were all low or average, thus there was no floristic indication for disturbances in the plots (*Endospermum malaccensis,* Euphorbiaceae: 2.3 trees/ha, *Dillenia obovata*, Dilleniaceae: 3.0 trees/ha, *Macaranga sp,* Euphorbiaceae: 0.7 trees/ha, *Campnosperma auriculatum*, Anacardiaceae: 1 tree/ha; compared to a mean of all tree species of 2.7 trees/ha, or the maximum density of 20.3 trees/ha of *Vatica pallida*, Dipterocarcpaceae).

Our comparison has shown differences between the forest in Siberut and other forests in Malesia in terms of an impoverishment of tree species richness, lower tree basal area and lower rattan density. Collectively, this may lead to a lower diversity and abundance of fruit resources, and this could possibly explain the large travel distances and the high amount of time devoted to traveling in Siberut macaques.

### Conservation and future of Siberut macaques

Based on the present results, we can give the following advice for future conservation action plans for Siberut macaques. First, it has to be taken into account that Siberut macaques need a much larger home range than any of the other primate species on Siberut. An appropriate conservation area would be an area large enough to sustain several groups to facilitate emigration processes and gene flow, and large enough to include sufficient fruit resources during seasons of low fruit availability. The area should consist of an intact, rather than fragmented, forest area as the dense, continuous forest was the most frequently used habitat type and was the basis of their food supply. Large forest trees in particular are important both as fruit resources and sleeping trees, as was already assumed by Whitten and Whitten (1982). Thus, selective logging of the large trees would immensely disturb their livelihood.

The future of Siberut macaques will unavoidably be closely connected to habitat degradation and loss. As macaques are generally omnivorous, they have the advantage of being able to adapt more easily to habitat changes than more specialized primates. However, as fruit constitutes a large percentage of their diet, they are likely to raid crops on farms if their original forest habitat does not supply enough fruit anymore, and this would increase conflicts with local people. In Siberut, local people traditionally hunt and trap primates, and Siberut macaques are caught in ground traps baited with sago, with which they can trap a whole group at once (pers. comm. by C. Abegg). Thus, Siberut macaques will become more vulnerable with increasing habitat loss and degradation.

The habitat of Siberut macaques is decreasing continuously. From the total land area of Siberut probably all covered with rainforest in the past, 87% was left in 1980 and only 60% in 2005 (2,400 km^2^,Table one in Whittaker 2006). From the remaining forest cover, 1,926 km^2^ are assigned to the Siberut National Park, but only 465 km^2^ are a protected no-use sanctuary zone where no hunting and logging is allowed (Fuentes1997/1997). A recent investigation has shown that the density of Siberut macaques in the national park is about three times lower than in the SCP area where this study was conducted (data from M. Quinten, unpublished), which indicates that the national park alone might not be enough to conserve Siberut macaques in the long-term. Although the status of Siberut macaques is not yet listed as critical, their population decreased from ~39,000 individuals in 1980 to 17,000-30,000 individuals in 2005 (Whittaker 2006) and if this decline continues, their future may soon be at risk.

## Methods

### Study area

**Figure 10:**
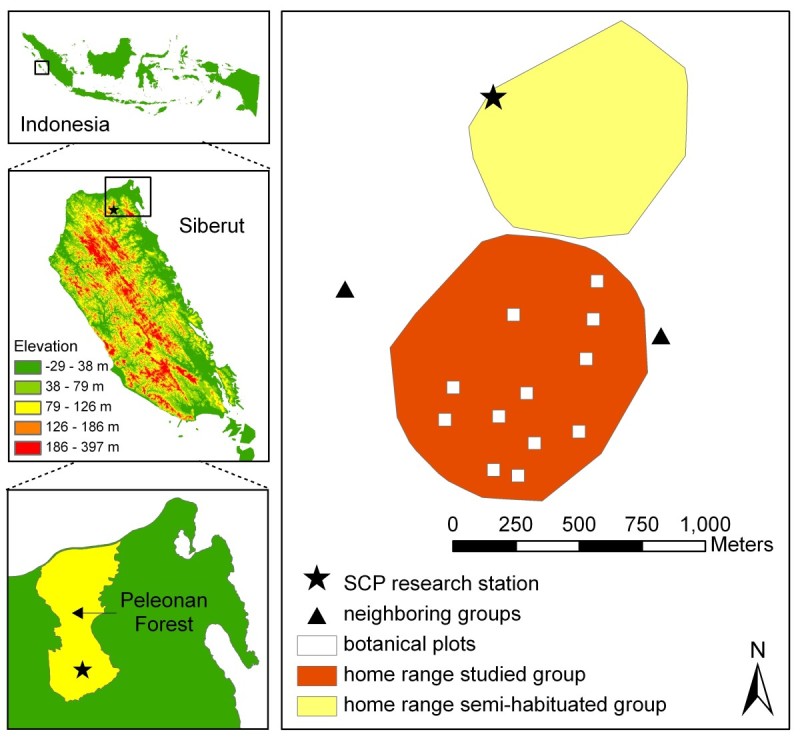
**Location of Siberut, the Peleonan Forest (rented by SCP for conservation purpose) and the study site (SCP research station).** The right map shows the 95% MCP home ranges of the studied group A (bottom), the semi-habituated group B (top) and locations of encounters with other neighboring groups of Siberut macaques. Small squares within the study group’s home range indicate the 12 botanical plots. Data source: 90 m digital elevation data come from Shuttle Radar Topography Mission (SRTM) by USGS/ NASA (http://srtm.csi.cgiar.org).

The study was conducted on Siberut Island, which comprises 4,030 km^2^ and a human population of ~25,000 people (Fuentes1997/1997;Whittaker 2006). Siberut has a strongly dissected, rugged landscape of numerous steep slopes and ravines (highest elevation: 384 m a.s.l.), and many rivers and streams (WWF 1980;Watanabe 1981). The island is covered by tropical lowland evergreen broadleaf rainforest (UNEP-WCMC classification) or tropical moist broadleaf forest (WWF classification). Different vegetation types can be distinguished: primary dipterocarp forest on high ridges (dominated by Dipterocarpaceae), primary mixed forest on slopes and lower hills (mixed composition of tree families with none being dominant), freshwater swamp forest, mangrove forest and Barringtonia forest on the West coast of Siberut (WWF 1980). The soil on Siberut is less fertile than on the Malay Peninsular (Whitten 1980;Whitten 1982a). The study was conducted at the field site of the Siberut Conservation Programme (SCP; www.siberut-island.org), situated within the Peleonan Forest in North Siberut (Figure 10). The Peleonan Forest comprises 4,500 ha rented by SCP for conservation purposes, surrounded by logging concessions and the Indian Ocean. It consists of undisturbed primary rainforest (i.e. with no signs of human impact) and some secondary forest at late successional stage. The climate is equatorial, with no seasonal changes in temperature. Temperature recorded during February 2010 and March 2011 ranged between 20.7°C and 35.2°C, with a monthly average of 25-27°C. There are only small seasonal changes in rainfall, and during our study, March was the driest month, and October to January the wettest period (max. rainfall per day: 150 mm/m^3^). Long-term climate data over 50 years show a mean annual rainfall of 3,601 mm at our study site, and every month of the year is perhumid, receiving at least 200 mm of rain (see Figure 1 in Erb et al.^2012^a).

### Study group

The study group (group A) was habituated from December 2008 until March 2010. Habituation was done by searching the group silently from sunrise to sunset with two to four teams simultaneously on six days per week. Once macaques were encountered, geographical position, subgroup size and activity was recorded. Beginning of 2010, at the end of the habituation period and when data collection started, group size was 29. There were 3 permanent adult males and 8 adult females in the group. Adult individuals were the main focus of this study and were all identified, except two of the adult females which were first seen at the end of the study. We assume that they were already part of the group throughout the data collection period, but were never reliably identified before as they might have stayed very peripheral and shy. Adults were defined as those individuals who were sexually fully mature, i.e. had large testes in males and visible nipples in females. By the end of the study period, during the mating season in January and February 2011, 3 juvenile females developed their first sexual swelling. From the juveniles, only a few individuals were identified, the rest was recorded as age-sex category during data collection.

As this group of Siberut macaques is the first one ever studied in detail, we report some information on the life history and demography. Infants are born with white fur and reddish facial skin, hands and feet. After few weeks, their fur coloration changes to juvenile/adult like dark brown coat with only small white fur patches left around the temples and a slightly lighter breast fur. At an age of about 2.5 months, the coloration change is complete. During the course of this study several births occurred (Mar.-Apr. 2009 3 birth, Sep.-Oct. 2009 4 birth (1 died), Jan. 2010 1 birth, Jul. 2010 1 birth (died at 2 month age)).

The studied group was surrounded by several other groups of conspecifics (Figure 10), including one group (group B) which was habituated from May to November 2009 and occasionally came to the research station when trees were fruiting there.

### Data collection

Group scan observations (scan sampling: Martin and Bateson 1993) were conducted from March 2010 until March 2011, from 6 am until latest 7:30 pm, by 1 to 3 observers simultaneously (69.3% by 1 observer, 27.0% by 2 observers, 3.7% by 3 observers). Scans were taken every full hour (sampling duration: 10 min) in the first month, and were changed to every 30 min at half and full hours for the rest of the time period (sampling duration: 5 min per scan). Data in March 2011 were too scarce and were omitted for some analyses. During group scans, the following data were recorded: time, identity of the monkeys, type of habitat (forest, canopy gap, windthrow area, swamp), relative and absolute height of the monkey in the forest, activity and in case they were feeding, the food item and species, as well as the GPS coordinates.

To calculate the monthly percentage of habitat use, we scored for each scan the habitat type used by the majority (>50%) of individuals. Canopy gap was defined as a small open area within the forest caused by a treefall. Windthrow area was a forest area hit by a heavy storm, which destroyed nearly all trees in that specific area.

Forest strata use was measured as the absolute forest height used by the monkeys (in 5 m steps) and the relative height used. Values for the whole group are based on the average of the values of each age-sex class (adult males, adult females, juveniles). The relative forest height describes the height of the monkey relative to the forest at a certain place in the forest, and was divided into 4 categories: ground (soil and leaf litter), lower-story (substrates on the ground, including fall-down trees, up to the mid-story), mid-story (either a tree that ends below the canopy at this place in the forest or the lower branches of a tree that make up the canopy) and canopy (the crown of the tree that makes up the canopy at this place in the forest).

The general activity of the monkeys at a scan time was classified as either traveling, resting, feeding (inserting food into the mouth, handling/ manipulating food; but not processing food which was already stored in the cheek pouch), foraging (searching for food), and social activities (allogrooming, mating, playing). Activity budget is given as the monthly average of 12 months, from March 2010 until February 2011.

Diet was based on the percentage of feeding time on different food items based on scan data. Food item categories were fruit, flower, arthropods, mushrooms, leaves, pith (soft core of palm stems), sap and shoot (young stems). The overall diet was calculated as the average over 12 months (Mar. 2010 – Feb. 2011). The dietary diversity index was calculated using the Shannon-Wiener index H’ (Pielou 1966;Krebs 1999). The index combines information on species richness as well as relative abundance. For calculation of H’, only food items of known species were used, which included fruit, flower, pith, sap and some of the leaves. Mushrooms and arthropods were not identified to species level and were thus not included in the index.

GPS points were recorded for 2,267 scans in a geographic coordinate system in a Lat/Lon format (Datum: WGS 84) and later converted into the projected coordinate system WGS 1984 UTM Zone 47 South. We only used GPS coordinates of permanent group members for home range analysis. Home range was calculated as Minimum Convex Polygon (MCP, Worton 1987;Harris et al. 1990;White and Garrott 1990;Börger et al. 2006) to allow comparison with older studies, and with Kernel methods using reference bandwidth *h*_*ref*_*,* which equaled 63.85 m (Silverman 1986;Worton 1989;Wand and Jones 1995;Worton 1995;Seaman and Powell 1997;Kenward 2001). We also applied the “ad hoc” bandwidth *h*_*ad hoc*_ (Berger and Gese 2007;Jaques et al. 2009;Kie et al. 2010), but results were similar to *h*_*ref*_ and are not reported. For MCP calculation (with “fixed means”), we used 4,839 unique point locations (duplicate fixes removed) and calculated 100% (the maximum area in which the group was ranging), 95% (full home range, reducing the outlier effect), and 50% MCPs (core home range). For kernel polygons, average GPS coordinates were calculated for each scan as the center of the group, as GPS points of single individuals per scan are not independent, resulting in 2,410 point locations. Points were jittered by ±0.5 m to avoid point duplicates, and kernels were constructed using fixed kernel approach, Gaussian (bivariate normal) kernels, a raster cell size of 20 m and a buffer of 25 m (the median distances to the group center). Although group center coordinates per scan were autocorrelated, we used all for kernel analysis because removing autocorrelation would also remove biologically significant information (Lair 1987;Reynolds and Laundre 1990;de Solla et al. 1999;Blundell et al. 2001;Cushman et al. 2005). We calculated the 95% contour as the full home range and the 50% contour as the core home range. Home range (and travel distance) calculations were done in ArcGIS® 9 (ArcEditor™ 9.3.1), using the Home Range Tools (HRT) extension (Version 1.1), except for the asymptote analysis of home range area, which was done with the extension HoRAE (Nov. 2011) in OpenJUMP 1.4.3.

The monthly average daily travel distance was calculated from group center coordinates per scan (see above). We only used days with ≥9 h observation time per day (called “full day follows”). Travel speed (m/h) is calculated as the travel distance per observation day divided by the observation time on that day. As the active period of the group was usually about 12 hours, from 6:30 am to 6:30 pm, this calculated travel speed was multiplied by 12 to obtain the daily travel distance. Straight line distances of groups in 30 min intervals can be different from actual travel distances of individuals (Isbell et al. 1999), so that we also report the average travel speed of single individuals. This is based on focal animal observations of all adult males and females, from August 2010 until February 2011, which aimed to be at least 30 min long (mean 33 min, max. 127 min). For analysis, we only used observations of at least 20 min.

For habitat analysis, we established 12 permanent botanical plots of 50 m x 50 m size (0.25 ha each) within the group’s home range (Figure 10), in which 25 subplots of 10 m x 10 m were nested. A total of 3 ha was sampled, covering 3.6% of the 95% kernel home range. Plots were distributed semi-randomly, while taking habitat variation, altitude and distance to rivers into account (top of hill: 1, slope: 2, slope/ riverine: 1, riverine/ level ground: 1, dry level ground: 6, edge of swamp: 1). Plots were mainly covering mixed forest, and to a low extent dipterocarp forest. We recorded all trees ≥10 cm dbh (diameter at breast height), all palm trees ≥10 cm dbh (*Oncosperma horridum*) or ≥5 cm dbh respectively (A*renga obtusifolia*, Pinanga sp.), all lianas (woody vines) and stranglers (strangler figs) ≥5 cm dbh and all rattan (i.e. climbing palms) longer than 5 m. They were marked, measured for dbh, and height (or length for lianas and rattan) was estimated by eye after training with a measuring tape. For trees with buttress roots or prop roots, dbh was measured ~20 cm above the rooting point. For clustering (rhizomatous) rattan and palm trees which produce multiple stems, we counted “apparent” genets, following Gerwing et al (2006). For the rhizomatous and very spiny palm tree *Oncosperma horridum*, we recorded the number of stems ≥10 cm dbh (important for basal area calculation), and for height and dbh, a mean was estimated. For rhizomatous rattan, dbh and length was measured for each stem separately. We collected two specimen per species with the help of a local plant specialist (31% of species with fruit and/or flower), and described, photographed and dried them in a self-made herbarium oven at 60-75°C in the field. They were identified (using Kooders 1913;Sinclair 1955;Kostermans 1969;Kostermans 1970;van Steenis 1972;Polunin 1988;van Balgooy 1997;van Balgooy 1998;de Wilde 2000;Symington et al. 2004;Yoneda 2004;Berg et al. 2005;Min et al. 2006;Soepadmo et al. 2007) and stored at the Herbarium ANDA of the Andalas University Padang, W-Sumatra. For analysis, we classified species into trees, palm trees, lianas, stranglers and rattan to enhance comparability between plots and other studies (Hadi et al. 2009b). The dbh distribution of trees from botanical plots is compared to 235 sleeping trees (Dec. 2008 – Mar. 2011) and to 73 feeding trees (Mar. 2010 – Mar. 2011; palm trees excluded). Basal area (m^2^) was calculated as 3.142 * (dbh in cm/ 200)^2^. We measured species diversity with the Simpson’s diversity index D, calculated as *D* = 1 - (*Σ n* * (*n* - 1)/*N* * (*N* - 1)), with n being the total number of individuals of a particular species, and N being the total number of individuals of all species. D ranges between zero (no diversity) and one (max. diversity). In addition, we also report the Shannon-Wiener diversity index.

Statistical analysis: Spearman rank correlations were conducted in Excel®, and p-values were based on 10,000 permutations. Mann-Whitney-U tests and Chi-Square tests were carried out in R 2.14.0© 2011. Species richness estimators, similarity indices and the Shannon-Wiener diversity index were calculated using EstimateS 8.2.0© 2009 (R.K. Colwell; http://purl.oclc.org/estimates). The species richness estimators ACE, ICE, Chao 1, Chao 2, Jack 1, Jack 2 and Bootstrap are reported as the mean of 10,000 randomizations, without replacement. For all analyses, we adopted an α-level of 0.05.

## Authors’ original submitted files for images

Below are the links to the authors’ original submitted files for images. Authors’ original file for figure 1 Authors’ original file for figure 2 Authors’ original file for figure 3 Authors’ original file for figure 4 Authors’ original file for figure 5 Authors’ original file for figure 6 Authors’ original file for figure 7 Authors’ original file for figure 8 Authors’ original file for figure 9 Authors’ original file for figure 10
