# Supplementary figures and images for: Ecology of an endemic primate species (Macaca siberu) on Siberut Island, Indonesia
Source: Springerplus. 2013 Mar 29;2:137. doi: 10.1186/2193-1801-2-137 (PMC3663992; doi:10.1186/2193-1801-2-137)

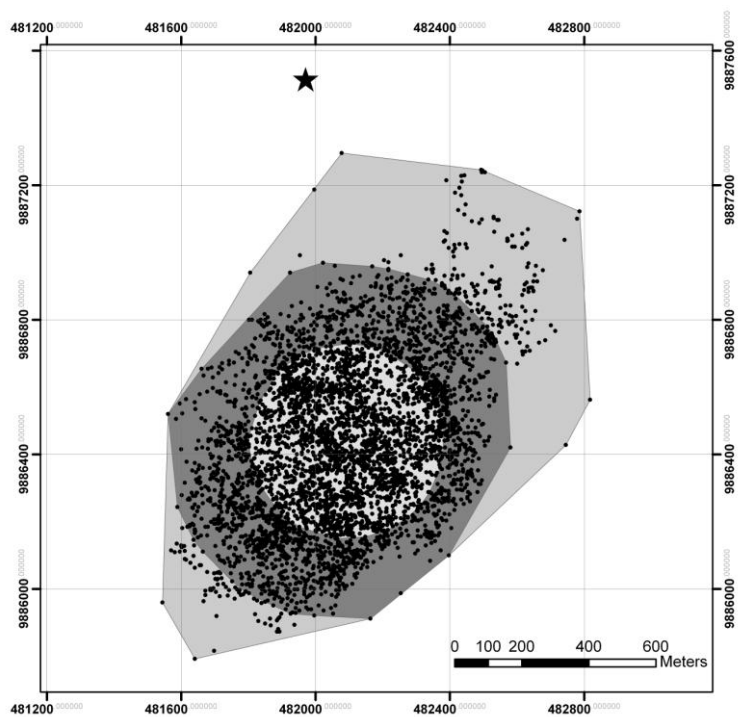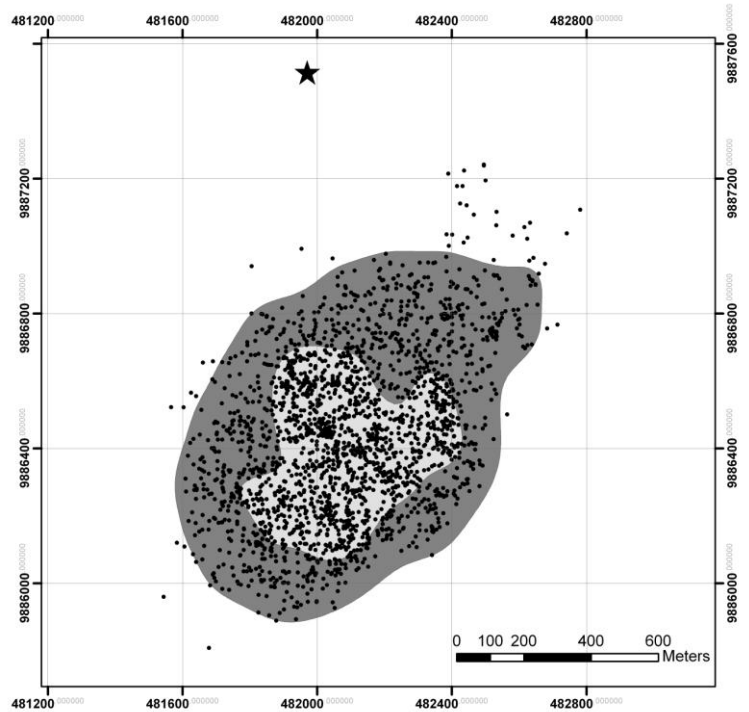

Supplement: Supplementary file 1 — Authors’ original file for figure 1 [file 40064_2012_274_MOESM1_ESM.pdf]

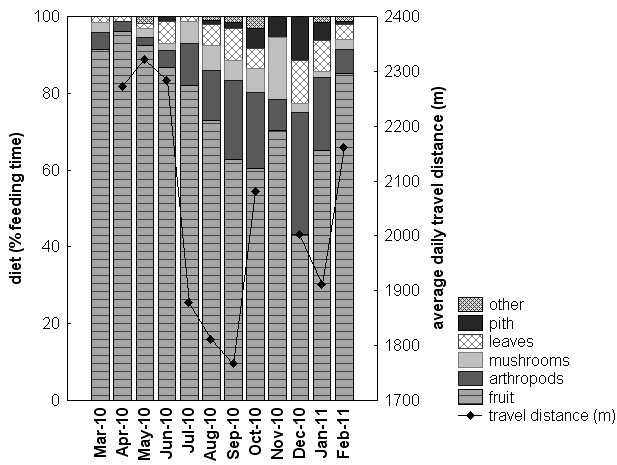

Supplement: Supplementary file 2 — Authors’ original file for figure 2 [file 40064_2012_274_MOESM2_ESM.jpeg]

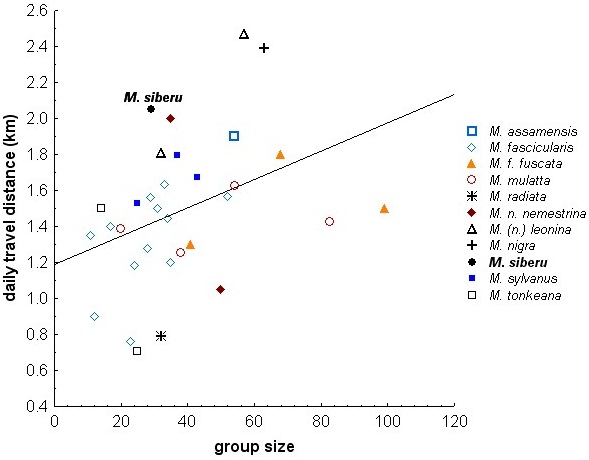

Supplement: Supplementary file 3 — Authors’ original file for figure 3 [file 40064_2012_274_MOESM3_ESM.jpeg]

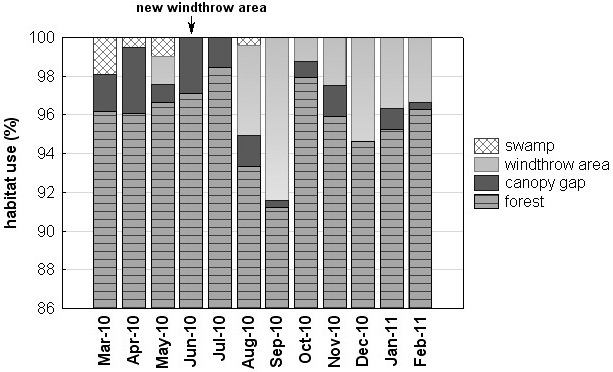

Supplement: Supplementary file 4 — Authors’ original file for figure 4 [file 40064_2012_274_MOESM4_ESM.jpeg]

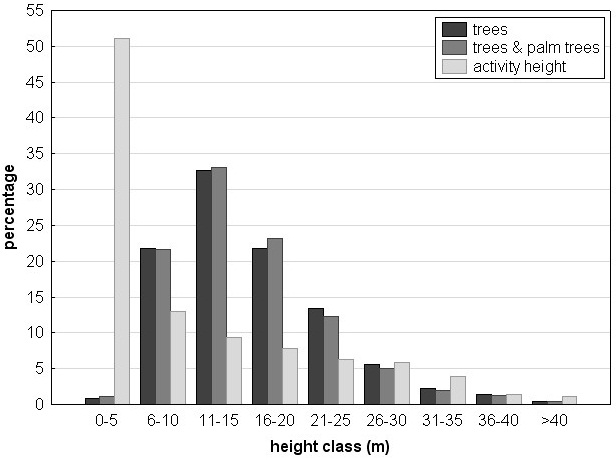

Supplement: Supplementary file 5 — Authors’ original file for figure 5 [file 40064_2012_274_MOESM5_ESM.jpeg]

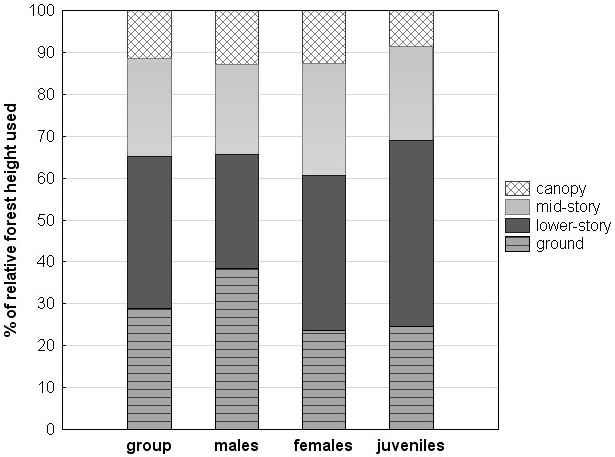

Supplement: Supplementary file 6 — Authors’ original file for figure 6 [file 40064_2012_274_MOESM6_ESM.jpeg]

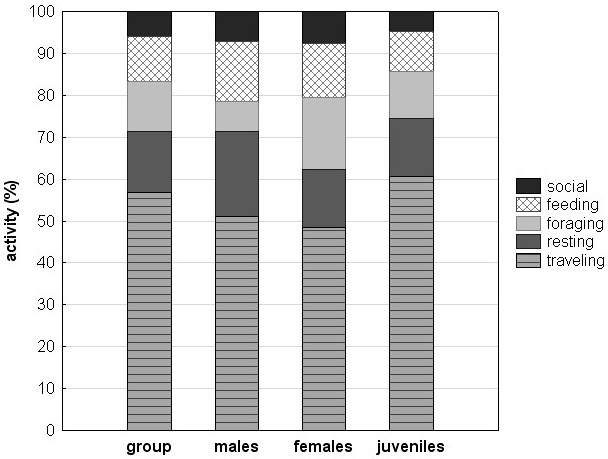

Supplement: Supplementary file 7 — Authors’ original file for figure 7 [file 40064_2012_274_MOESM7_ESM.jpeg]

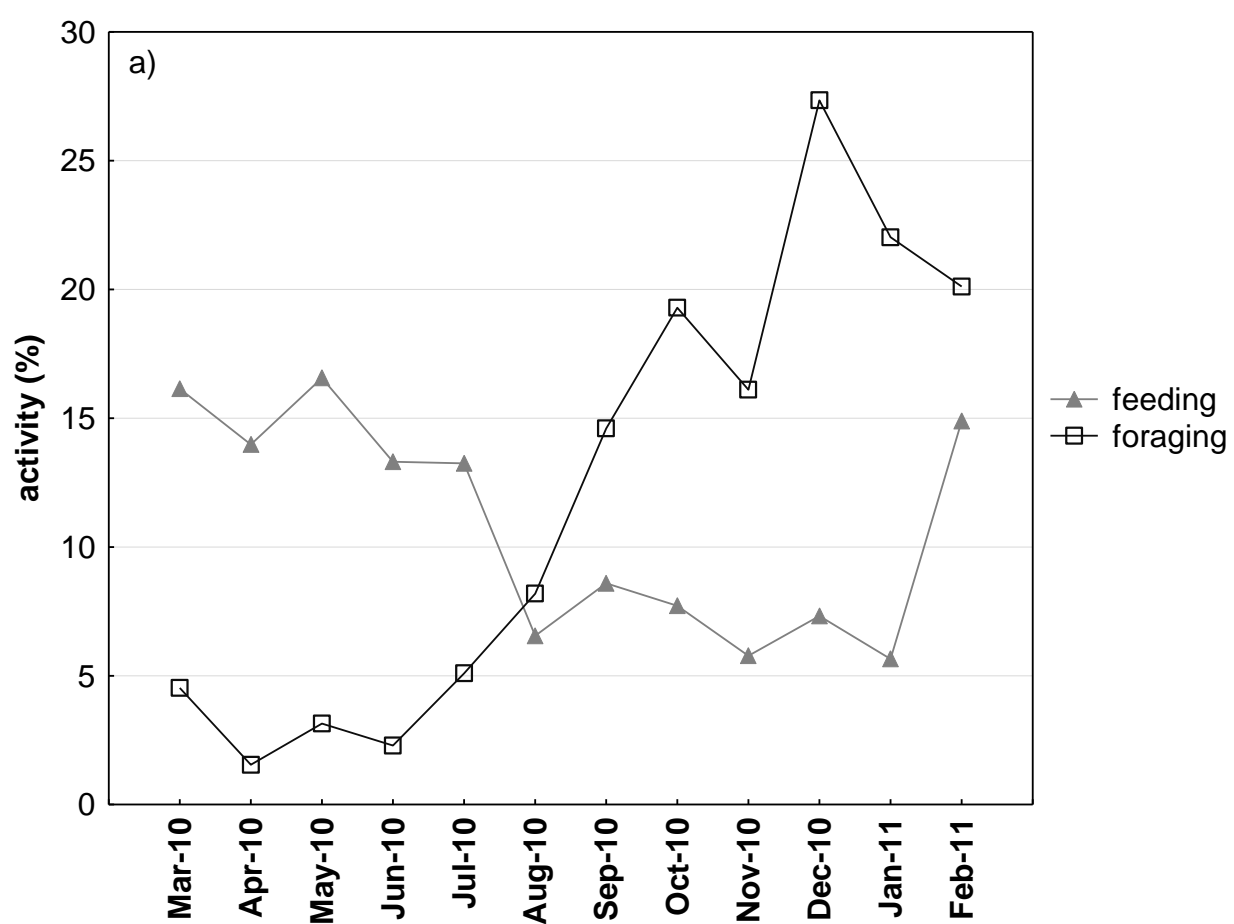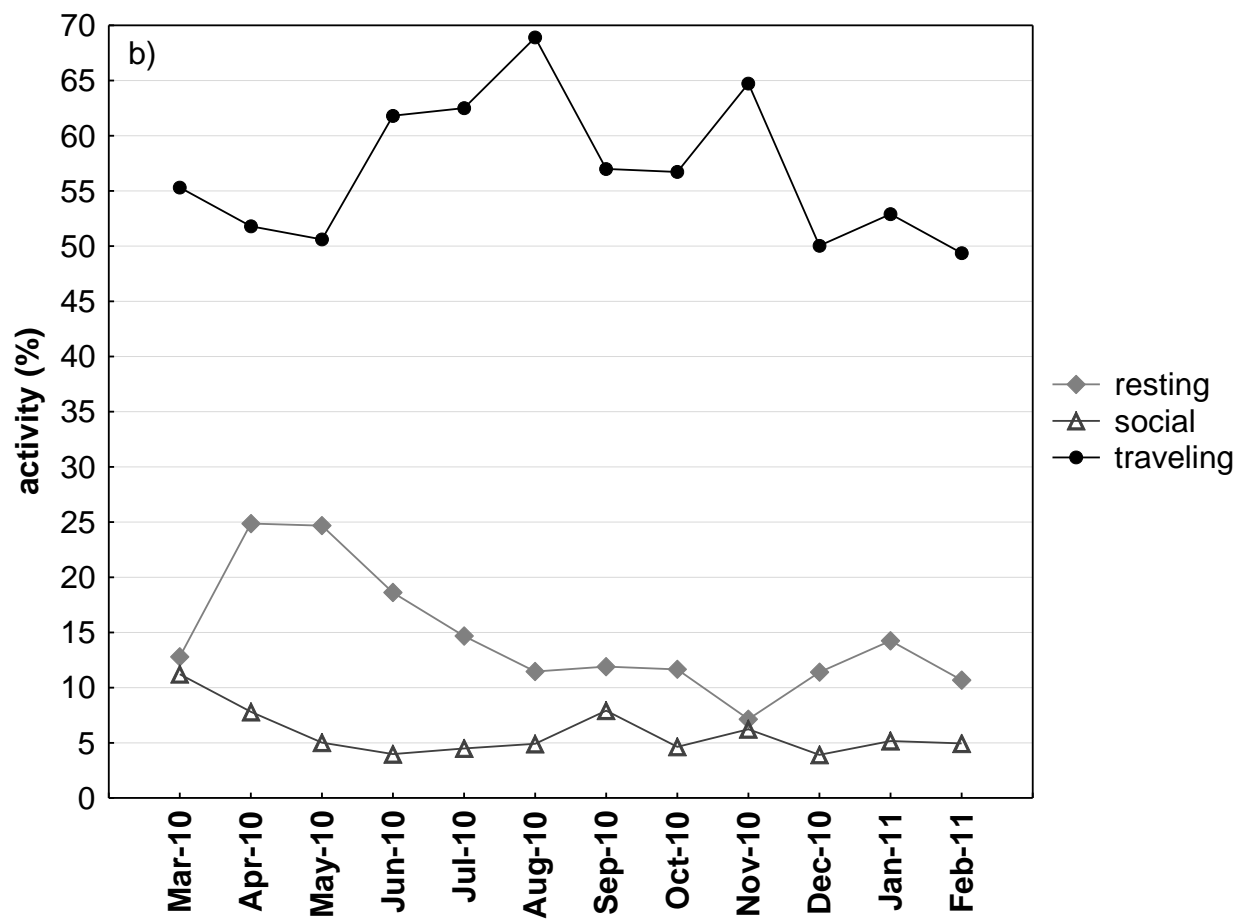

Supplement: Supplementary file 8 — Authors’ original file for figure 8 [file 40064_2012_274_MOESM8_ESM.pdf]

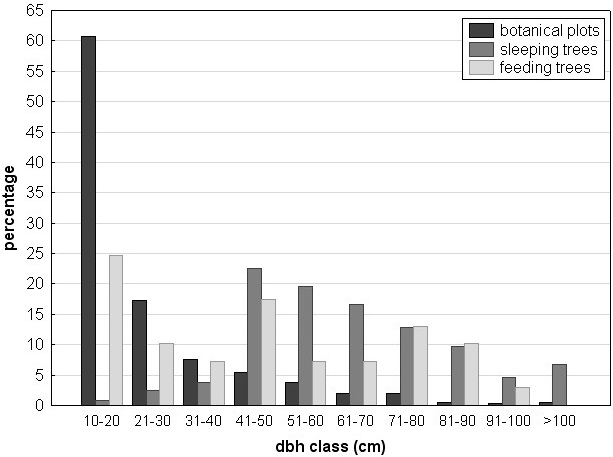

Supplement: Supplementary file 9 — Authors’ original file for figure 9 [file 40064_2012_274_MOESM9_ESM.jpeg]

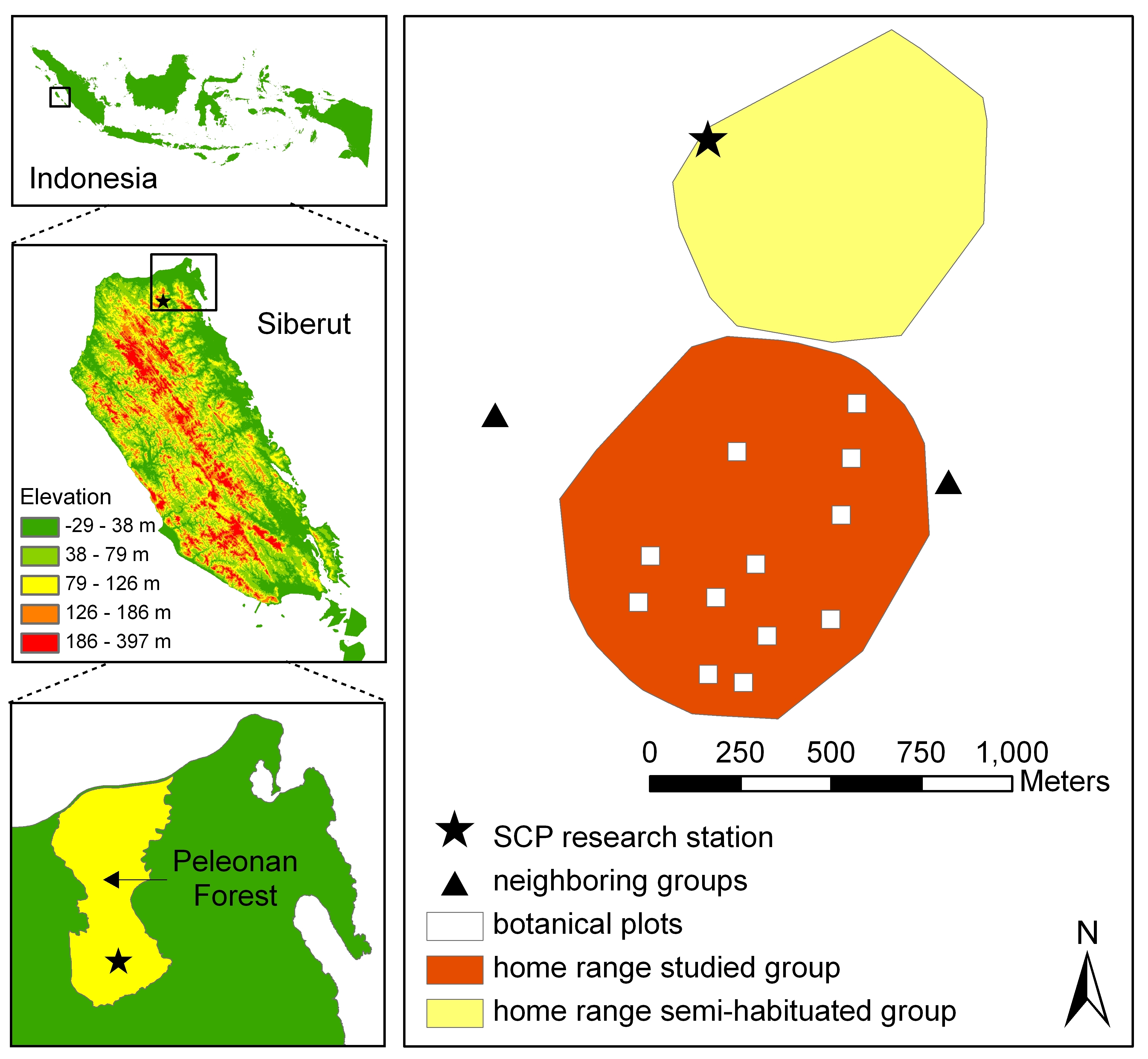

Supplement: Supplementary file 10 — Authors’ original file for figure 10 [file 40064_2012_274_MOESM10_ESM.jpeg]
